# Patterns of person‐centred communications in public HIV clinics: a latent class analysis using the Roter interaction analysis system

**DOI:** 10.1002/jia2.26119

**Published:** 2023-07-06

**Authors:** Njekwa Mukamba, Chanda Mwamba, Salil Redkar, Marksman Foloko, Kasapo Lumbo, Herbert Nyirenda, Debra L. Roter, Musunge Mulabe, Anjali Sharma, Sandra Simbeza, Kombatende Sikombe, Laura K. Beres, Jake M. Pry, Katerina Christopoulos, Charles B. Holmes, Elvin H. Geng, Izukanji Sikazwe, Carolyn Bolton‐Moore, Aaloke Mody

**Affiliations:** ^1^ Centre for Infectious Disease Research in Zambia Lusaka Zambia; ^2^ University of Washington St. Louis St. Louis Missouri USA; ^3^ Department of Health Behaviour and Society Johns Hopkins Bloomberg School of Public Health Baltimore Maryland USA; ^4^ Department of International Health Johns Hopkins Bloomberg School of Public Health Baltimore Maryland USA; ^5^ California Department of Public Health Richmond California USA; ^6^ Department of Medicine University of California San Francisco San Francisco California USA; ^7^ Centre for Global Health and Quality Georgetown University Medical Center Washington DC USA; ^8^ Department of Medicine University of California San Francisco San Francisco California USA

**Keywords:** patient−provider communication, Roter interaction analysis system (RIAS), patient experience, latent class analysis, HIV, retention in care

## Abstract

**Introduction:**

Poor client−provider communication is a critical barrier to long‐term retention in care among people living with HIV. However, standardized assessments of this key metric are limited in Africa. We used the Roter Interaction Analysis System (RIAS) to quantitatively characterize patterns of person‐centred communication (PCC) behaviours in Zambia.

**Methods:**

We enrolled pairs of people living with HIV making routine HIV follow‐up visit and their providers at 24 Ministry of Health‐facilities supported by the Centre for Infectious Disease Research in Zambia in Lusaka province between August 2019 and November 2021. Client−provider encounters were audio‐recorded and coded using RIAS by trained research staff. We performed latent class analysis to identify interactions with distinctive patterns of provider PCC behaviours (i.e. rapport building, person‐centred counselling, PCC micropractices [e.g. brief empathy statements], assessing barriers to care, shared decision‐making and leveraging discretionary power) and compared their distribution across client, provider, interaction and facility characteristics.

**Results:**

We enrolled 478 people living with HIV and 139 providers (14% nurses, 73.6% clinical officers, 12.3% were medical officers). We identified four distinct profiles: (1) “Medically Oriented Interaction, Minimal PCC Behaviours” (47.6% of interactions) was characterized by medical discussion, minimal psychosocial/non‐medical talk and low use of PCC behaviours; (2) “Balanced Medical/Non‐medical Interaction, Low PCC Behaviours” (21.0%) was characterized by medical and non‐medical discussion but limited use of other PCC behaviours; (3) “Medically Oriented Interaction, Good PCC Behaviours” (23.9%) was characterized by medically oriented discussion, more information‐giving and increased use of PCC behaviours; and (4) “Highly person‐centred Interaction” (7.5%) was characterized by both balanced medical/non‐medical focus and the highest use of PCC behaviours. Nurse interactions were more likely to be characterized by more PCC behaviours (i.e. Class 3 or 4) (44.8%), followed by medical officers (33.9%) and clinical officers (27.3%) (*p* = 0.031). Longer interactions were also more likely to integrate more PCC behaviours (*p* < 0.001).

**Conclusions:**

PCC behaviours are relatively uncommon in HIV care in Zambia, and often limited to brief rapport‐building statements and PCC micropractices. Strengthening PCC, such as shared decision‐making and leveraging discretionary power to better accommodate client needs and preferences, may be an important strategy for improving the quality in HIV treatment programmes.

## 1 INTRODUCTION

The encounter with healthcare providers is a pivotal moment in the client experience, but negative experiences with providers have remained one of the critical drivers of loss to follow‐up among people living with HIV [1, 2, 3, 4, 5, 6, 7, 8, 9, 10, 11, 12, 13]. Attention to the client experience is emerging as a key global public health priority as a means of providing whole‐person care, fostering lifelong engagement, and improving treatment and quality of life‐related outcomes [14, 15]. Emerging efforts to improve the person‐centredness of care delivery (e.g. differentiated service delivery) have primarily focused on changing the care infrastructure [16, 17, 18], but relatively less emphasis has been placed on targeting the underlying nature of client−provider interactions and integration of person‐centred communication (PCC) behaviours (e.g. shared decision‐making, attention to empathy, open‐ended questions and using discretionary power to accommodate client needs) [15, 19, 20, 21, 22, 23, 24, 25]. A deeper understanding of these interactions and patterns, frequencies and typologies of client−provider communication behaviours can help inform strategies to improve the person‐centredness and quality of care delivery, and, ultimately, long‐term outcomes in public health HIV facilities [7, 10, 15, 19, 26, 27, 28, 29, 30].

Standardized assessments of client−provider communication that seek to quantify and characterize patterns of communications can yield insights into the frequency and types of different communication behaviours that help contextualize the experiences and gaps reported by clients and providers. Qualitative evidence suggests that rude behaviour and scolding drive people out care and also discourage them from returning after lapses, but it is not immediately clear how prevalent and frequent these behaviours are [1, 2, 3, 4, 5, 6, 7, 8, 9, 10, 11, 12, 13]. Furthermore, communication can often be hierarchical, directive and dominated by the provider with limited efforts to elicit input from clients regarding preferences or anticipated challenges with care/treatment, which can further exacerbate other ongoing barriers (e.g. competing obligations and stigma) [13, 21, 22, 23, 29, 31, 32, 33, 34, 35, 36]. Due to different roles and responsibilities in overburdened health systems, these behaviours and interaction dynamics may also manifest differently across healthcare worker (HCW) cadres [21, 22, 31, 37, 38, 39]. Systematically characterizing the different patterns of client−provider communication can thus reveal valuable insights about its current state in public health HIV clinics and help to build a better understanding of the road forward for delivering truly person‐centred care.

In this study, we used the Roter interaction analysis system (RIAS) to systematically parse and characterize patterns of PCC behaviours in Zambia. RIAS is a validated method for assessing and quantifying aspects of client−provider communication that has been used globally across diverse settings [29, 32, 33, 39, 40, 41, 42, 43, 44, 45]. We then used latent class analysis (LCA) to identify distinctive profiles of communication during routine HIV follow‐up visits in public health facilities in Zambia and assessed how they vary across client, provider, interaction and facility characteristics.

## 2 METHODS

### 2.1 Study population and setting

We enrolled dyads of adults living with HIV (18 years or older) making a routine visit for HIV care and their HIV care providers for that visit between August 2019 and November 2021 from 24 facilities in Lusaka province. Facilities were run by the Ministry of Health and received technical assistance from the Centre for Infectious Disease Research in Zambia (CIDRZ). Each facility provided similar HIV treatment services and cared for populations requiring similar levels of care (site selection was driven primarily by proximity), although catchment area demographics, staffing, HCW cadre representation and facility infrastructure could vary (Table S1).

All care providers conducting routine HIV monitoring visits at facilities were offered enrolment and consented at study initiation during staff meetings. This included nurses, clinical officers (similar to physician assistant) and medical officers (similar to a medical doctor). Due to the task‐shifting for Antiretroviral Therapy (ART) scale‐up, each HCW cadre is trained to provide appropriate care for routine HIV follow‐up (i.e. visits scheduled every 3−6 months for monitoring) in public health HIV facilities in Zambia.

On days that previously enrolled providers were in clinic, people living with HIV presenting to facilities for a routine HIV follow‐up visit were conveniently sampled from the waiting room and consented prior to entering the consultation room (typically 20−30 minutes beforehand). As providers and clients were consented independently, some enrolled providers may not have seen an enrolled client due to normal staff rotations and transfers. These procedures provided for a sample that was representative of routine public health HIV services in Zambia. Those presenting for more specialized or focused visits (e.g. enhanced adherence counselling visits, tuberculosis or maternal and child health) were not included. Due to COVID‐19, all study activities and recruitment were paused from 24 March to 16 June 2020, which also coincided with when a majority of healthcare disruptions occurred [46, 47]. After this period, healthcare and in‐person visits began to normalize, but individuals were often provided longer refills to reduce facility traffic; this practice continued even after the initial COVID‐19 lockdown period [46]. The official “lockdown” period lasted until 30 August 2020 in Zambia.

### 2.2 Procedure and measurements

After obtaining consent, we audio‐recorded the routine HIV follow‐up interaction between clients and their providers. We used remote‐controlled audio‐recorders that were discretely placed in provider rooms to remotely start and stop recording when an enrolled client entered and left the consultation room. These procedures allowed for unobtrusive recording in order to mitigate changes to client or provider communication behaviours due to awareness of being observed (i.e. Hawthorne effect).

Audio‐recorded visits were linked to the participant and visit data from the national electronic health record (EHR) used in routine HIV care in Zambia. This EHR contains socio‐demographic (e.g. age, sex and clinic site), clinical (date of ART initiation and WHO stage) and visit history (dates and scheduled appointment) measurements. Individuals were linked using identification numbers; visits were linked if they were within 5 days of each other to account for minor discrepancies in data entry into the EHR.

### 2.3 Analyses

Audio‐recordings of client−provider interactions were coded using RIAS. RIAS is a quantitative method of coding designed to parse and classify client and provider communication into operationally defined codes and standardized dimensions [40, 41, 42, 45]. It has been previously validated across a wide range of clinical and cultural settings, and quantifies aspects of communication that have been associated with outcomes, such as satisfaction and adherence [29, 32, 33, 39, 40, 41, 42, 43, 44]. The RIAS method involves coders assigning each utterance (i.e. a statement representing a complete thought) made by the client or provider into one of 37 mutually exclusive and exhaustive categories based on standardized definitions, such as question‐asking (e.g. open vs. closed), information‐giving (e.g. clinical vs. psychosocial), socio‐emotional communication (e.g. empathy statements and rapport building) and provider:client speech ratio [40, 41, 42]. We also generated study‐specific codes to use with the RIAS method that identified occurrences of PCC behaviours emphasized in our previous formative work and PCC frameworks (Tables 1 and S2) [1, 4, 15, 19, 21, 23, 25]. These included rapport building, PCC micropractices (i.e. brief PCC behaviours, such as offering encouragement, checking for understanding), assessing barriers to HIV care, person‐centred counselling (i.e. counselling incorporating PCC behaviours like empathy, validation), use of shared decision‐making and leveraging discretionary power (i.e. using discretion in decision‐making to better meet client needs) [1, 4, 15, 19, 20, 21, 22, 23, 25].

**Table 1:** Description of Roter interaction analysis system (RIAS) codes

RIAS composite code	Description
Partnership (Doc)	Percent of statements that are partnering statements by provider (e.g. asking opinion, checking understanding and positive statement)
Medical question (Doc)	Percent of statements that are questions about medical/therapeutic topics
Psychosocial question (Doc)	Percent of statements that are questions about psychosocial/lifestyle topics
Medical information (Doc)	Percent of statements that are information/counselling about medical/therapeutic topics
Psychosocial information (Doc)	Percent of statements that are information/counselling about psychosocial/lifestyle topics
Psychosocial‐medical ratio	Ratio of psychosocial to medical questions/statement
Provider speech ratio (Doc)	Percent of utterances from provider
Open‐ended question (Doc)	Percent of questions that were open‐ended (vs. close‐ended)
Rapport building	Percent of interactions with at least one provider statement meant to build rapport with the client
PCC micropractice	Percent of interactions with at least one small client‐centred practice by provider (e.g. asking for feedback, providing encouragement and explaining decision rationale)
Barriers to HIV care	Percent of interactions where barriers to HIV care were assessed by provider
Person‐centred counselling	Percent of interactions where principles from PCC were integrated into counselling (e.g. empathy, offering encouragement and asking for understanding)
Shared decision‐making	Percent of interactions where providers used shared decision‐making (i.e. jointly decided care plan with input from client)
Discretionary power	Percent of interactions where providers leveraged discretionary power to better align care with client needs

Abbreviations: PCC, Person‐centred communication; Doc, Doctor.

Coding was conducted by three CIDRZ qualitative researchers who were fluent in local languages (i.e. Nyanja, Bemba and English). Coders were trained in the RIAS method during a 3‐day intensive workshop held in August 2019 in Lusaka, Zambia, and demonstrated a high degree of inter‐coder reliability during training (Pearson correlation 0.8).

### 2.4 Statistical analyses

We performed LCA to identify and characterize interactions with distinctive profiles of PCC behaviours using data generated from RIAS coding. LCA is a well‐established data‐driven method to empirically identify groups that have distinctive patterns in their data that are not readily observed or identified [48]. In our LCA, we included variables on the frequency of different types of speech (e.g. medical/psychosocial questions or information giving, partnering statements), ratios of different types of speech (e.g. provider‐client utterance ratio, psychosocial‐medical utterance ratio, percent open‐ vs. closed‐ended questions) and the PCC‐specific RIAS codes (Tables 1 and S2). After systematically tested model fit with differing number of classes, we selected a final model that was optimized for fit and parsimony—using Akaike's Information Criterion and Bayesian Information Criterion—and interpretability—using contextual knowledge [48, 49]. From this final data‐driven model, we then estimated the probability of each client−provider interaction belonging to a specific latent class (i.e. communication profile) based on their observed patterns (i.e. estimated posterior probabilities) and assigned them to the latent class to which they were most likely to belong (i.e. the maximal probability rule) [48, 49]. We assessed the adequacy and fit of the final model and group assignment using several established metrics [48, 49]. Lastly, each profile was named to descriptions of communication patterns observed.

We describe RIAS patterns for client−provider interactions overall as well as by HCW cadre. To identify associations with client, provider, interaction, and facility characteristics and communication profiles, we describe the distribution of latent classes (i.e. communication profiles) and assess variability across these characteristics. For both, we used Kruskal−Wallis and Pearson chi‐square tests, as appropriate. As a sensitivity analysis, we used multinomial logistic regression to assess the association between communication profiles and client, provider, interaction and facility‐level characteristics after also *adjusting* for these characteristics. Lastly, as an exploratory analysis among visits that could be linked to the EHR, we assessed whether the communication profile at the current visit was associated with being more than 30 days late for the next appointment using mixed‐effects Poisson regression with robust variances (Supplementary Appendix).

Additional details on our statistical analyses are provided in the Supplementary Appendix.

All analyses were conducted using Stata (Version 17.0, College Station, Texas). This descriptive substudy represents a secondary analysis embedded within a larger parent stepped‐wedge cluster‐randomized trial—Leveraging Person‐Centred Public Health (PCPH) to improve HIV outcomes in Zambia (PACTR202101847907585). Sample size calculations were for the primary trial; there were no formal calculations for this secondary analysis. The study was approved by the University of Zambia Biomedical Research Ethics Committee (UNZABREC) (March 2019) in Zambia and institutional review boards at the University of Alabama, Birmingham School of Medicine (June 2019) and Washington University in St. Louis (July 2019) in the United States. This paper was prepared according to STROBE guidelines.

## 3 RESULTS

### 3.1 Client, provider, interaction and facility characteristics

**Figure 1:**
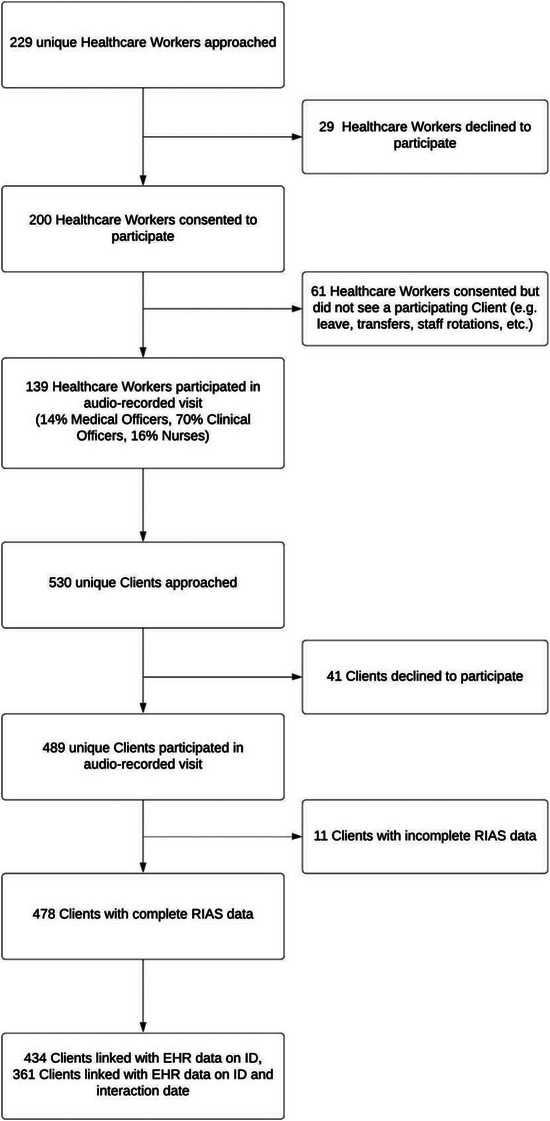
Participant flowchart. Abbreviations: EHR, electronic health record; RIAS, Roter interaction analysis system

Between 1 August 2019 and 1 November 2021, we enrolled and audio‐recorded interactions between 478 people living with HIV and 139 providers from 24 facilities (Figure 1). 14% of HCWs were nurses, 73.6% were clinical officers and 12.3% were medical officers. Among clients, 62.6% were female, and most were between 30 and 50 years old. 52.7% of interactions occurred in Nyanja, 31.8% in English and 15.5% in Bemba. The median length of interactions was 7.8 minutes (IQR 5.5–11.9). 17.8% of interactions occurred prior to the COVID‐19 pandemic (1 August 2019–31 March 2020), 11.9% occurred during the initial lockdown period (1 April 2020–31 August 2020) and 70.3% of interactions occurred afterwards (1 September 2020–1 November 2021) (Table 2).

**Table 2:** Client, provider, interaction and facility characteristics (*N* = 478)

Client	Overall (*N* = 478)	Nurse (*n* = 67)	Clinical officer (*n* = 352)	Medical officer (*n* = 59)	*p*‐value
Sex, *n* (%)					
Female	299 (62.6%)	41 (61.2%)	220 (62.5%)	38 (64.4%)	0.93
Male	179 (37.4%)	26 (38.8%)	132 (37.5%)	21 (35.6%)	
Age, *n* (%)					
18−30 years	89 (18.6%)	10 (14.9%)	71 (20.2%)	8 (13.6%)	0.76
31−40 years	160 (33.5%)	23 (34.3%)	116 (33.0%)	21 (35.6%)	
41−50 years	137 (28.7%)	20 (29.9%)	98 (27.8%)	19 (32.2%)	
>50 years	59 (12.3%)	11 (16.4%)	40 (11.4%)	8 (13.6%)	
Missing	33 (6.9%)	3 (4.5%)	27 (7.7%)	3 (5.1%)	
Marital status, *n* (%)					
Single	67 (14.0%)	12 (17.9%)	44 (12.5%)	11 (18.6%)	0.40
Married	244 (51.0%)	30 (44.8%)	187 (53.1%)	27 (45.8%)	
Divorced	50 (10.5%)	10 (14.9%)	32 (9.1%)	8 (13.6%)	
Widowed	34 (7.1%)	6 (9.0%)	25 (7.1%)	3 (5.1%)	
Missing	83 (17.4%)	9 (13.4%)	64 (18.2%)	10 (16.9%)	
Education, *n* (%)					
None	17 (3.6%)	4 (6.0%)	12 (3.4%)	1 (1.7%)	0.012
Primary	115 (24.1%)	14 (20.9%)	85 (24.1%)	16 (27.1%)	
Secondary	233 (48.7%)	31 (46.3%)	169 (48.0%)	33 (55.9%)	
University	24 (5.0%)	10 (14.9%)	11 (3.1%)	3 (5.1%)	
Missing	89 (18.6%)	8 (11.9%)	75 (21.3%)	6 (10.2%)	
Time since enrolment in care, *n* (%)					
<6 months	42 (8.8%)	7 (10.4%)	30 (8.5%)	5 (8.5%)	0.99
6 months−1 year	44 (9.2%)	7 (10.4%)	31 (8.8%)	6 (10.2%)	
1−2 years	53 (11.1%)	8 (11.9%)	38 (10.8%)	7 (11.9%)	
2−5 years	114 (23.8%)	16 (23.9%)	81 (23.0%)	17 (28.8%)	
> 5 years	190 (39.7%)	26 (38.8%)	143 (40.6%)	21 (35.6%)	
Missing	35 (7.3%)	3 (4.5%)	29 (8.2%)	3 (5.1%)	
Enrolment WHO Stage, *n* (%)					
WHO Stage 1	197 (41.2%)	27 (40.3%)	147 (41.8%)	23 (39.0%)	0.37
WHO Stage 2	54 (11.3%)	12 (17.9%)	38 (10.8%)	4 (6.8%)	
WHO Stage 3	42 (8.8%)	4 (6.0%)	31 (8.8%)	7 (11.9%)	
WHO Stage 4	3 (0.6%)	0 (0.0%)	2 (0.6%)	1 (1.7%)	
Missing	182 (38.1%)	24 (35.8%)	134 (38.1%)	24 (40.7%)	
**Provider**					
Sex, *n* (%)					
Female	236 (49.4%)	51 (76.1%)	159 (45.2%)	26 (44.1%)	<0.001
Male	242 (50.6%)	16 (23.9%)	193 (54.8%)	33 (55.9%)	
Provider type, *n* (%)					
Nurse	67 (14.0%)	–	–	–	–
Clinical officer	352 (73.6%)	–	–	–	
Medical officer	59 (12.3%)	–	–	–	
**Interaction**					
Interaction language, *n* (%)					
Nyanja	252 (52.7%)	32 (47.8%)	198 (56.2%)	22 (37.3%)	0.012
English	152 (31.8%)	28 (41.8%)	97 (27.6%)	27 (45.8%)	
Bemba	74 (15.5%)	7 (10.4%)	57 (16.2%)	10 (16.9%)	
Sex concordance (client—provider), *n* (%)					
Female—female	159 (33.3%)	33 (20.7%)	109 (68.5%)	17 (10.7%)	<0.001
Female—male	140 (29.3%)	8 (5.7%)	111 (79.3%)	21 (15%)	
Male—female	77 (16.1%)	18 (23.3%)	50 (64.9%)	9 (11.7%)	
Male—male	102 (21.3%)	8 (7.8%)	82 (80.3%)	12 (11.7%)	
Time period, *n* (%)					
01 Aug 2019−31 Mar 2020	85 (17.8%)	14 (20.9%)	55 (15.6%)	16 (27.1%)	0.11
01 Apr 2020−30 Aug 2020	57 (11.9%)	4 (6.0%)	47 (13.4%)	6 (10.2%)	
01 Sept 2020−30 Nov 2021	336 (70.3%)	49 (73.1%)	250 (71.0%)	37 (62.7%)	
**Facility**					
Facility type^a^, *n* (%)					
Small clinic	98 (20.5%)	19 (28.4%)	79 (22.4%)	0 (0.0%)	<0.001
Medium clinic	180 (37.7%)	16 (23.9%)	136 (38.6%)	28 (47.5%)	
Large clinic	81 (16.9%)	8 (11.9%)	55 (15.6%)	18 (30.5%)	
Hospital‐based clinic	119 (24.9%)	24 (35.8%)	82 (23.3%)	13 (22.0%)	
ART integration^b^, *n* (%)					
Non‐integrated	319 (66.7%)	53 (79.1%)	212 (60.2%)	54 (91.5%)	<0.001
Integrated	159 (33.3%)	14 (20.9%)	140 (39.8%)	5 (8.5%)	
Client:provider ratio^c^, median (IQR)	328 (223, 547)	249 (176, 605)	328 (223, 547)	266 (184, 496)	0.47

^a^ Small clinic−0–2500 clients; Medium clinic−2500–10,000 clients; Large clinic—>10,000 clients; Hospital‐based clinic—outpatient clinic based at facility that also provided inpatient hospital services.

^b^ Non‐integrated—ART services provided during standalone clinic session; Integrated—ART services integrated with other primary care services.

^c^ Number of clients in a facility clinic population over number of providers offering ART services at that facility, averaged quarterly.

Abbreviations: ART, antiretroviral therapy; WHO, World Health Organization.

Interactions, in general, focused primarily on medical topics with providers speaking a majority of the time. Open‐ended questions were very rare compared to close‐ended questions (although slightly more common among clinical officers). Clinical officers asked more questions (particularly medical), whereas nurses provided more information statements. PCC behaviours, such as rapport building, and PCC micropractices were more common, but more complex behaviours, such as using shared decision‐making and discretionary power, were rarer. Nurses spent more time with clients compared to clinical and medical officers (Tables 3 and S3).

**Table 3:** Client−provider communication across healthcare worker cadre using RIAS (*N* = 478)

	Overall (*N* = 478)	Nurse (*n* = 67)	Clinical officer (*n* = 352)	Medical officer (*n* = 59)	*p*‐value
Partnership (Doc), mean (SD)	0.24 (0.12)	0.23 (0.11)	0.25 (0.12)	0.21 (0.11)	0.055
Medical question (Doc), mean (SD)	0.20 (0.11)	0.15 (0.09)	0.21 (0.11)	0.18 (0.09)	<0.001
Psychosocial question (Doc), mean (SD)	0.05 (0.05)	0.05 (0.04)	0.05 (0.05)	0.06 (0.05)	0.20
Medical information (Doc), mean (SD)	0.29 (0.16)	0.32 (0.18)	0.28 (0.16)	0.29 (0.19)	0.14
Psychosocial information (Doc), mean (SD)	0.04 (0.06)	0.06 (0.08)	0.04 (0.05)	0.05 (0.06)	0.014
Psychosocial‐medical ratio, mean (SD)	0.16 (0.14)	0.18 (0.16)	0.15 (0.13)	0.20 (0.14)	0.018
Provider speech ratio, mean (SD)	0.59 (0.07)	0.59 (0.07)	0.59 (0.07)	0.59 (0.07)	0.85
Open‐ended questions (Doc), mean (SD)	0.02 (0.04)	0.01 (0.02)	0.03 (0.04)	0.01 (0.02)	<0.001
Rapport building, *n* (%)	425 (88.9%)	61 (91.0%)	313 (88.9%)	51 (86.4%)	0.71
PCC micropractice	320 (66.9%)	52 (77.6%)	234 (66.5%)	34 (57.6%)	0.055
Barriers to HIV care, *n* (%)	196 (41.0%)	22 (32.8%)	144 (40.9%)	30 (50.8%)	0.12
Person‐centred counselling, *n* (%)	198 (41.4%)	33 (49.3%)	146 (41.5%)	19 (32.2%)	0.15
Shared decision‐making, *n* (%)	114 (23.8%)	16 (23.9%)	86 (24.4%)	12 (20.3%)	0.79
Discretionary power, *n* (%)	45 (9.4%)	9 (13.4%)	30 (8.5%)	6 (10.2%)	0.44
Interaction length in minute, median (IQR)	7.8 (5.5−11.9)	12.3 (7.0−18.1)	7.4 (5.2−11.0)	8.5 (5.8−12.1)	<0.001

Abbreviations: SD, standard deviation; IQR, interquartile range; Doc, doctor.

### 3.2 Description of latent classes

**Figure 2:**
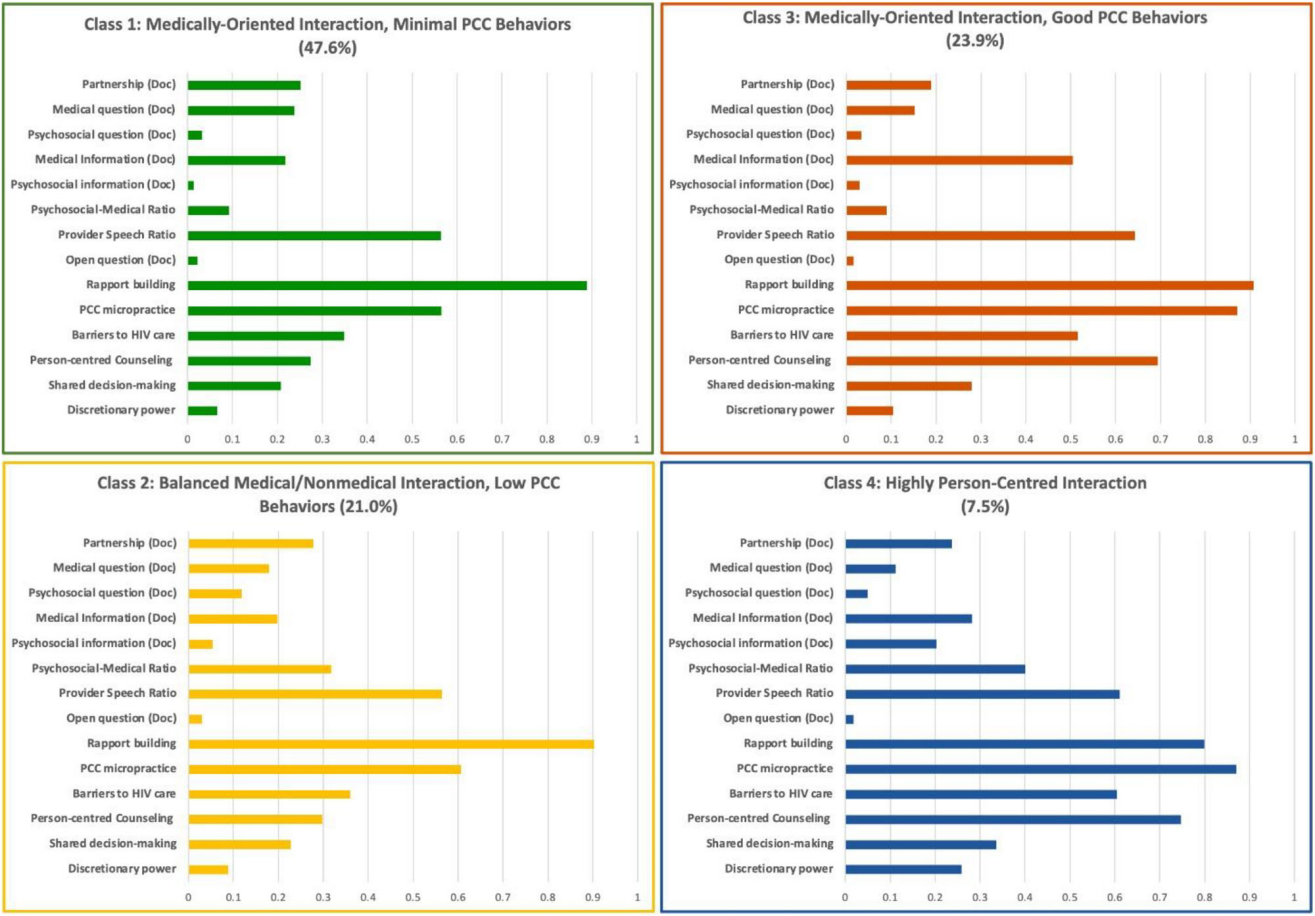
Profiles of client–provider communication using Roter interaction analysis system (RIAS) (*N* = 478). Communication profiles are based on latent class models using measurements from RIAS coding. Abbreviations: Doc, doctor; PCC, person‐centred communication

We selected the model with four latent classes (i.e. profiles of client−provider interactions) based on model fit and interpretability (Figure 2, Tables S4 and S5). 47.6% (95% CI 41.5–53.8%) of interactions were characterized by discussion predominately around medical topics, minimal discussion of psychosocial topics and relatively low use of PCC behaviours, such as shared decision‐making and leveraging discretionary power (“Medically Oriented Interaction, Minimal PCC Behaviours”). The second class (“Balanced Medical/Non‐medical Interaction, Low PCC Behaviours” group, 21.0% of interactions [95% CI 16.5–26.4%]) was characterized by more balance between medical and psychosocial topics, but still low use of PCC behaviours. The third class (“Medically Oriented Interaction, Good PCC Behaviours” group, 23.9% of interactions [95% CI 19.1–29.4%]) was characterized again by predominately medically oriented discussion, but greater use of PCC behaviours, including integrating PCC principles into counselling, using more PCC micropractices, assessing barriers to HIV care and using shared decision‐making. The final class (“Highly person‐centred Interaction” group) represented only 7.5% (95% CI 5.2–10.6%) of interactions but was characterized by discussion of both medical and psychosocial topics and the highest use of PCC behaviours. Several diagnostic metrics indicated that this final model had very good fit and separation between classes (Table 4).

**Table 4:** Metrics of adequacy and fit of latent class model

Class	Class size	Group average posterior probability	Odds ratio for correct classification	Estimated group distribution using maximal probability rule	Estimated group distribution based on initial model	Entropy
Medically oriented interaction, minimal PCC behaviours	237	0.918	12.4	0.496	0.476	0.87
Balanced medical/non‐medical interaction	95	0.937	56.0	0.199	0.210	
Medically oriented interaction, good PCC behaviours	111	0.92	36.5	0.232	0.239	
Highly person‐centred interaction	35	0.961	306	0.073	0.075	

Note: Good model fit indicated by (1) average posterior probability greater than 0.7 for each group; (2) odds ratio of correct classification greater than 5 for each group; (3) close correspondence between the estimated group distribution based on using posterior probabilities and the maximal probability rule compared with the estimated group distribution from the initial model; and (4) an entropy greater than 0.8.

Abbreviation: PCC, Person‐centred communication.

### 3.3 Characteristics associated with latent classes

There were a few notable associations between client, provider, interaction, and facility characteristics and different communication profiles. Interactions integrating more PCC behaviours (“Medically Oriented, Good PCC Behaviours” and “Highly person‐centred Interaction” groups) were more frequent with nurses, while less person‐centred interactions (“Medically Oriented Interaction, Minimal PCC Behaviours” and “Balanced Medical/Non‐medical Interaction, Low PCC Behaviours” groups) were more common with clinical officers. Profiles with more PCC behaviours were also more common at hospital‐based and small clinics compared to medium and large clinics as well as integrated compared to non‐integrated ART clinics. Third, the increased length of the interaction was associated with profiles with more PCC behaviours. In general, interactions with younger age groups (18–30 and 31–40 year‐olds) tended to integrate more PCC behaviours compared to older clients. There was no association with client or provider sex nor sex concordance and PCC behaviour profiles. Last, integration of PCC behaviours increased over time, with no change in trend during COVID‐19 (Table 5). These patterns were similar in adjusted sensitivity analyses (Table S6).

**Table 5:** Client, provider, interaction and facility characteristics by latent class (*N* = 478)

	Medically oriented interaction, minimal PCC behaviours (*n* = 237)	Balanced medical/non‐medical interaction, low PCC behaviours (*n* = 95)	Medically oriented interaction, good PCC behaviours (*n* = 111)	Highly person‐centred interaction (*n* = 35)	*p*‐value
**Client**					
Sex, *n* (%)					
Female	147 (49.1%)	65 (21.7%)	69 (23.0%)	18 (6.0%)	0.35
Male	90 (50.2%)	30 (16.7%)	42 (23.4%)	17 (9.5%)	
Age, *n* (%)					
18−30 years	37 (41.6%)	17 (19.1%)	29 (32.6%)	6 (6.7%)	0.025
31−40 years	67 (41.9%)	35 (21.9%)	41 (25.6%)	17 (10.6%)	
41−50 years	75 (54.7%)	32 (23.4%)	21 (15.3%)	9 (6.6%)	
>50 years	34 (57.6%)	8 (13.6%)	16 (27.1%)	1 (1.7%)	
Marital status, *n* (%)					
Single	30 (44.8%)	16 (23.9%)	16 (23.9%)	5 (7.5%)	0.64
Married	122 (50%)	47 (19.3%)	57 (23.4%)	18(7.4%)	
Divorced	21 (42%)	13 (26%)	9 (18%)	7 (14%)	
Widowed	19 (55.9%)	4 (11.8%)	9 (26.5%)	2 (5.9%)	
Education, *n* (%)					
None	10 (58.8%)	1 (5.9%)	5 (29.4%)	1 (5.9%)	0.097
Primary	59 (51.3%)	29 (25.2%)	18 (15.7%)	9 (7.8%)	
Secondary	96 (41.2%)	52 (22.3%)	65 (27.9%)	20 (8.6%)	
University	13 (54.2%)	1 (4.2%)	8 (33.3%)	2(8.3%)	
Time since enrolment in care, *n* (%)					
<6 months	19 (45.2%)	9 (21.4%)	10 (23.8%)	4 (9.5%)	0.85
6 months−1 year	20 (45.5%)	9 (20.5%)	11 (25%)	4 (9.1%)	
1−2 years	26 (49.1%)	12 (22.6%)	13 (24.5%)	2 (3.8%)	
2−5 years	48 (42.1%)	21 (18.4%)	35 (30.7%)	10 (8.8%)	
> 5 years	98 (51.6%)	41 (21.6%)	38 (20%)	13 (6.8%)	
Enrolment WHO Stage, *n* (%)					
WHO Stage 1	102 (51.8%)	36 (18.3%)	47 (23.9%)	12 (6.1%)	0.56
WHO Stage 2	26 (48.2%)	8 (14.8%)	15 (27.8%)	5 (9.3%)	
WHO Stage 3	24 (57.1%)	8 (19.1%)	6 (14.3%)	4 (9.5%)	
WHO Stage 4	2 (66.7%)	0 (0%)	0 (0%)	1 (33.3%)	
					
**Provider**					
Sex, *n* (%)					
Female	106 (44.9%)	52 (22%)	59 (25%)	19 (8.1%)	0.25
Male	131 (54.1%)	43 (17.8%)	52 (21.5%)	16 (6.6%)	
Provider type, *n* (%)					
Nurse	26 (38.8%)	11 (16.4%)	23 (34.3%)	7 (10.5%)	0.031
Clinical officer	189 (53.7%)	67 (19%)	74 (21%)	22 (6.3%)	
Medical officer	22 (37.3%)	17 (28.8%)	14 (23.7%)	6 (10.2%)	
**Interaction**					
Interaction language, *n* (%)					
Nyanja	128 (50.8%)	50 (19.8%)	51 (20.2%)	23 (9.1%)	0.12
English	67 (44.1%)	30 (19.7%)	47 (30.9%)	8 (5.3%)	
Bemba	42 (56.8%)	15 (20.3%)	13 (17.6%)	4 (5.4%)	
Sex concordance (client—provider), *n* (%)					
Female—female	75 (47.2%)	35 (22%)	39 (24.5%)	10 (6.3%)	0.37
Female—male	72 (51.4%)	30 (21.4%)	30 (21.4%)	8 (5.7%)	
Male—female	31 (40.3%)	17 (22.1%)	20 (26%)	9(11.7%)	
Male—male	59 (57.8%)	13 (12.8%)	22 (21.6%)	8 (7.8%)	
Time period, *n* (%)					
01 Aug 2019−31 Mar 2020	64 (75.3%)	14 (16.5%)	3 (3.5%)	4 (4.7%)	<0.001
01 Apr 2020−30 Aug 2020	29 (50.9%)	12 (21.1%)	14 (24.6%)	2 (3.5%)	
01 Sept 2020−30 Nov 2021	144 (42.9%)	69 (20.5%)	94 (28%)	29 (8.6%)	
Interaction length, minute, median (IQR)	7.0 (4.9−10.1)	7.48 (5.8−13.6)	9.4 (6.7−12.7)	11.2 (7.1−17.8)	<0.001
**Facility**					
Facility type^a^, *n* (%)					
Small clinic	48 (49.0%)	14 (14.3%)	29 (29.6%)	7 (7.1%)	0.008
Medium clinic	105 (58.3%)	30 (16.7%)	37 (20.6%)	8 (4.4%)	
Large clinic	37 (45.7%)	24 (29.6%)	15 (18.5%)	5 (6.2%)	
Hospital‐based clinic	47 (39.5%)	27 (22.7%)	30 (25.2%)	15 (12.6%)	
					
ART integration^b^, *n* (%)					
Non‐integrated	145 (45.5%)	64 (20.1%)	85 (26.6%)	25 (7.8%)	0.037
Integrated	92 (57.9%)	31 (19.5%)	26 (16.4%)	10 (26.6%)	
Client:provider ratio^c^, median (IQR)	276 (184−517)	276 (176−517)	328 (223−605)	421 (223−605)	0.27

Note: Percentages are calculated across rows to represent the distribution of latent class across client, provider, interaction and facility characteristics.

^a^ Small clinic: 0–2500 clients; Medium clinic: 2500–10,000 clients; Large clinic: >10,000 clients; Hospital‐based clinic: Outpatient clinic based at facility that also provided inpatient hospital services.

^b^ Non‐integrated: ART services provided during standalone clinic session; Integrated: ART services integrated with other primary care services.

^c^ Number of clients in a facility clinic population over number of providers offering ART services at that facility, averaged quarterly.

Abbreviations: ART, antiretroviral therapy; PCC, person‐centred communication; IQR, interquartile range; WHO, World Health Organization

### 3.4 Association between latent class and missing the next visit

Interactions during the COVID‐19 lockdown period and longer interactions were associated with a higher likelihood of being more than 30 days late for the next visit. There were few other client, provider, interaction or facility characteristics associated with being late, including provider communication profile (Table S7).

## 4 DISCUSSION

We identified four distinctive client−provider communication profiles in public HIV clinics in Zambia: 47.6% were predominately medically oriented with minimal PCC behaviours; 21.0% had a balance of medical and psychosocial discussion, but still low use of PCC; 23.9% were predominately medically oriented but had high use of PCC behaviours; and 7.5% demonstrated very high use of PCC behaviours, including shared decision‐making, use of discretionary power or integrating PCC principles into their counselling. Interactions with nurses and those that were longer tended to incorporate more PCC behaviours. These results provide deeper insights into the frequency and patterns of communication behaviours between clients and providers, and offer an important window into one of the key determinants of the client experience in public HIV clinics in Zambia.

Our study uses standardized procedures to parse and quantify communication behaviours during routine HIV monitoring visits in Zambia. Poor client−provider communication and interactions (e.g. rude behaviour and scolding) have been identified as a key determinant of the client experience and retention in care in these settings [1, 2, 3, 4, 5, 6, 7, 8, 9, 10, 11, 12, 13]. Our assessment extends and complements this existing evidence by characterizing the frequencies and typologies of these known gaps between client−provider communication and what clients desire [1, 7, 10, 21, 23, 25, 32]. We note that a majority of interactions focus primarily on medical topics, although a small but noticeable percentage of interactions give significant attention to psychosocial topics. Open‐ended questions were relatively infrequent compared to closed‐ended questions. Second, we find that more complex person‐centred behaviours, such as shared decision‐making, leveraging discretionary powers and integrated person‐centred practices into counselling, are still quite rare in public HIV clinics in Zambia. This may be a manifestation of the time and cognitive/emotional effort often required for these behaviours, which can be limited in overburdened facilities [31, 37, 50]. Nevertheless, the use of shared decision‐making and discretionary power can facilitate alignment of care delivery with clients’ needs and preferences, and thus may have meaningful impacts on the client experience [7, 15, 19, 20, 21, 22, 23, 25, 32]. Third, we find that providers practice rapport building (e.g. greetings and welcoming statements) and PCC micropractices with high frequency and that overtly negative interactions were also rare, which aligns with prior studies using client satisfaction surveys [10, 11, 12, 51]. Still, even occasional lapses (approximately 10% of interactions lacked any rapport‐building statements in our study) will cumulatively expose a substantial proportion of individuals over their care journey to a potentially negative interaction that could trigger a lapse in care. Lastly, these communication patterns, generally, fairly consistent across client, provider, interaction and facility characteristics (with some notable exceptions), and the totality of these findings also align remarkably well with client−provider communication patterns that have been previously identified, even across very different clinical and cultural settings [32, 34, 40, 42, 43, 45]. Still, it will be key to contextualize and validate these findings further, particularly to understand how these profiles reflect client experiences and capture what is relevant and desirable to them in our setting.

Patterns of communication appeared to differ across HCW cadres. Nurses tended to spend more time with clients and have interactions characterized by more person‐centred behaviours (and to a lesser extent medical officers). This is in contrast to clinical officers who had shorter interactions and fewer interactions classified with person‐centred behaviours. These differences may in part be attributable to our finding that nurses spent more time providing information or counselling as opposed to question‐asking (which was more frequent among clinical and medical officers). Moreover, differences in the hierarchy between clients and providers may have influenced these communication patterns [13, 21, 22, 23, 31, 32, 33, 34]. Importantly, it should also be recognized that these patterns may relate to the underlying reason for the visit and staffing at different facilities, rather than behaviours attributable to the cadre itself. For example, we found an association between longer visits and an increased likelihood of being late for the next visit, but this may have been driven by an increased complexity or challenges faced by those clients. Also, although we did not find clear evidence of this in our study, in Zambia, medical and clinical officers may sometimes be tasked to see individuals with more complex disease compared to their nurse colleagues, altering the nature of the interaction. Thus, it is critical to understand the primary drivers of these different patterns of communication behaviours (e.g. higher quality communication behaviour vs. nature of interaction vs. facility culture and climate) so that the appropriate improvement efforts can be targeted and prioritized.

Communication between client and provider is complex—varying across roles, purpose, setting and circumstances—and this dimensionality needs to be considered in strategies to improve the client experience and person‐centredness of care delivery. Negative HCW interactions impact long‐term retention in HIV care [1, 2, 3, 4, 5, 6, 7, 8, 9, 10, 11, 12], so it is imperative that health systems continuously foster the awareness and skills in providers for improving the care experience [14, 15, 23, 29, 30, 34]. Integrating skills to nurture trust and confidence throughout HCW training may help PCC behaviours become normalized skills at an earlier stage. Our findings suggest that providers do frequently use rapport‐building and brief person‐centred behaviours, but there is likely a need to prioritize more open‐ended questions and attention to psychosocial factors to facilitate more holistic discussions. Moreover, increased use of shared decision‐making and discretionary power—such as identifying convenient return dates and medication refill durations—could allow care delivery to more flexibly meet clients where they are at and avoid precipitating future care lapses [20, 21, 22, 23]. This substudy was nested within the larger *PCPH* study—which sought to assess a multi‐component implementation strategy comprised of training and mentoring on principals of person‐centred care, systematic audit‐and‐feedback of the patient experience and a small facility‐level incentive for improvements. Although the increase in PCC behaviours over time could have been related to this implementation strategy, this substudy is not able to isolate the cause of these changes away from secular trends due to COVID‐19 or a changing healthcare environment. Nevertheless, these skills will be critical in aiding public health HIV care delivery in Africa to mature beyond often protocolized and algorithmic care to more personalized approaches to public health [15, 17, 52, 53]. Furthermore, linking provider skills training with systematic measurement and feedback of relevant experience metrics (e.g. client‐reported outcomes and observed communication behaviours) will also provide more robust guidance on what strategies are needed to better the pivotal interactions between clients and their providers.

There are several limitations of our study. First, these profiles of client−provider communication were generated using a data‐driven approach and only provide a descriptive perspective on communication. Although model diagnostics indicated a very good fit, further efforts to validate these profiles against other measures of communication, particularly clients’ own assessments of communication, are needed. Nevertheless, our findings do align with previous research on this topic [10, 11, 21, 25, 31, 32, 34, 35, 40, 42, 43, 45], and generate contextual insights—such as the frequency of different behaviours and provider speaking dominance—that are not commonly captured. Second, differences in communication profiles may be explained by important elements in the interaction outside of what were able to capture (e.g. reason for visit and facility culture). Still, we did not identify substantial differences across measured client, provider, interaction and facility characteristics. Third, we did not assess interactions as clients were triaged or in the waiting room and the RIAS coding system may not have captured all the relevant dimensions of communication during consultations (even though specifically captured PCC behaviours identified as relevant during formative work [e.g. discretionary power, assessing barriers to HIV care and shared decision‐making]). Thus, measures may have been limited in their abilities to quantitatively capture the relationship between communication and missing the subsequent visit in exploratory analyses. Fourth, HCW or client behaviour may have been affected by knowing they were being observed (i.e. Hawthorne effect). We did, however, seek to minimize this bias as much as possible by consenting providers far beforehand and using remote‐controlled audio‐recorders. Fifth, we were unable to link some of the clients and visits to the EHR. Lastly, our sample size may have been too small to identify more nuanced differences, particularly given the known heterogeneity across facilities. Still, the variability in our sample was representative of HIV care in Zambia.

## 5 CONCLUSIONS

We used novel methods to quantitatively parse and characterize distinctive patterns of provider communication behaviours in public HIV clinics in Zambia. We identified four unique interaction profiles that varied in the degree to which they integrated PCC behaviours and were distributed across HCW cadres. These findings provide a nuanced characterization of the frequency and typologies of client−provider communication in the Zambian settings and highlight behaviours (e.g. use of shared decision‐making and discretionary power, reducing provider speech dominance) that may be strengthened to improve the client experience, care quality and long‐term engagement in public health HIV programmes.

## COMPETING INTERESTS

All the authors declare that they have no competing interests.

## AUTHORS’ CONTRIBUTIONS

AM and EHG conceived the study. NM, AM, SR, AS, LKB, HN, MF, KL and CM conducted the analysis and interpretation of data. NM coordinated the data collection. NM and AM wrote the first draft of the manuscript. AS, SR, LKB, DLR, CM, KC, IS, CB‐M, CBH and EHG provided critical feedback to enhance the intellectual content of the manuscript. All authors read and approved the final manuscript.

## FUNDING

This study was funded by the Bill & Melinda Gates Foundation (Grant number INV‐010563) and the National Institutes of Health (KL2 TR002346 to AM and K24 AI134413 to EHG). The funders had no role in study design, data collection, data analysis, data interpretation or writing of the report.

## DISCLAIMER

The authors alone are responsible for the views expressed in this publication and these do not necessarily represent the decision or stated policy of the Centre for Infectious Disease Research in Zambia (CIDRZ). The content is solely the responsibility of the authors and does not necessarily represent the official views of the Bill & Melinda Gates Foundation or the National Institutes of Health.

## Supporting information


**Supplementary Information**: Sensitivity and Exploratory Analyses and Tables S1 to S7

## Data Availability

The transcripts and datasets generated and/or analysed during this qualitative study are not publicly available due to privacy provisions but are available from the corresponding author on approval from the authorizing institutional review board.
