# Supplementary material for: Patterns of person‐centred communications in public HIV clinics: a latent class analysis using the Roter interaction analysis system
Source: J Int AIDS Soc. 2023 Jul 6;26(Suppl 1):e26119. doi: 10.1002/jia2.26119 (PMC10323315; doi:10.1002/jia2.26119)
Supplement: Supplementary file 1 — Supplementary Information: Sensitivity and Exploratory Analyses and Tables S1 to S7 [file JIA2-26-e26119-s001.docx]

***Patterns of Person-centred communications in public HIV clinics: A Latent Class Analysis using the Roter Interaction Analysis System (RIAS)***

**SUPPLEMENTAL APPENDIX**

**Sensitivity and Exploratory Analyses**……………………....……..…………………....……..……………….…….2

**Table S1:** Facility Characteristics and Staffing……………………....……..…..……………….……...………...……3

**Table S2:** Description of RIAS Categories and Abbreviations ……………………………………………….………4

**Table S3.** RIAS Across Characteristics**………………………………..**…………………………………..……………5

**Table S4.** Goodness-of-Fit Statistics for Latent Class Models with Different Number of Classes.……...………11

**Table S5:** Item Response Probabilities for Latent Classes…………………………………………………….……12

**Table S6.** Predictive Margins of Latent Class Distribution Across Client, Provider, Interaction, and Facility Characteristics from Multinomial Logistic Regression**…………….**..………………………...………………………13

**Table S7.** Association between Client-Provider Communication and Being Late to Next Visit by 30 days…….16

### Sensitivity and Exploratory Analyses

In a sensitivity analysis, we used multinomial regression to assess the association between communication profiles and client, provider, interaction, and facility-level characteristics after also adjusting for these characteristics. This analysis was meant to assess for any potential confounding between the various characteristics that could bias our primary descriptive and unadjusted results. We first ran a multinomial regression for predictors of class membership that included all relevant client, provider, interaction, and facility characteristics. We then used post-estimation commands to estimate the predictive margins of the expected distribution of latent classes stratified across each characteristic.

In an exploratory analysis, among visits that could be linked to the EHR, we also sought to assess whether latent class membership was associated with whether individuals were more than 30 days late for their next appointment. We performed unadjusted and adjusted mixed-effects Poisson regression with robust variances and to estimate risk ratios from binary outcomes [1-3]. Adjusted models included latent class membership as well as client, provider, interaction, and facility characteristics; facility was also included as a random effect.

To address missingness in some variables (e.g., client age, WHO stage, time in care, marital status, and education), we employed multiple imputation by chained equations for both of these analyses (n=20) [4].

### Table S1: Facility Characteristics and Staffing (n=24)

| Facility | Clinic Type^a^ | ART Clinic Integration Status^b^ | Total Staff Average | Average Medical Officer Staffing | Average Clinical Officer Staffing | Average Nursing Staffing | Average Client to Provider Ratio^c^ |
| --- | --- | --- | --- | --- | --- | --- | --- |
| 1 | Small | Non-Integrated | 5.8 | 0.3 | 1.3 | 4.3 | 39 |
| 2 | Hospital-based | Non-Integrated | 5.7 | 0.0 | 1.0 | 4.7 | 222 |
| 3 | Medium | Non-Integrated | 17.8 | 0.6 | 3.1 | 14.2 | 184 |
| 4 | Medium | Non-Integrated | 6.0 | 0.4 | 1.7 | 3.9 | 517 |
| 5 | Medium | Non-Integrated | 16.2 | 1.0 | 2.3 | 12.9 | 276 |
| 6 | Medium | Non-Integrated | 11.2 | 0.7 | 1.7 | 8.9 | 501 |
| 7 | Hospital-based | Non-Integrated | 11.7 | 0.7 | 2.3 | 8.7 | 992 |
| 8 | Hospital-based | Non-Integrated | 20.7 | 1.0 | 3.0 | 16.7 | 421 |
| 9 | Hospital-based | Non-Integrated | 11.0 | 1.3 | 2.7 | 7.0 | 99 |
| 10 | Medium | Integrated | 7.2 | 1.0 | 1.3 | 4.8 | 738 |
| 11 | Medium | Medium Clinic | 13.3 | 0.4 | 1.6 | 11.3 | 249 |
| 12 | Medium | Non-Integrated | 24.9 | 1.0 | 4.3 | 19.6 | 266 |
| 13 | Small | Non-Integrated | 2.7 | 0.0 | 1.3 | 1.3 | 176 |
| 14 | Large | Non-Integrated | 14.8 | 0.7 | 3.0 | 11.4 | 496 |
| 15 | Large | Integrated | 23.2 | 1.0 | 4.0 | 18.3 | 255 |
| 16 | Large | Non-Integrated | 14.2 | 0.3 | 3.0 | 10.8 | 693 |
| 17 | Small | Non-Integrated | 2.6 | 0.0 | 1.0 | 1.6 | 482 |
| 18 | Small | Integrated | 12.7 | 0.0 | 2.8 | 9.9 | 44 |
| 19 | Medium | Integrated | 5.5 | 0.3 | 2.1 | 3.2 | 877 |
| 20 | Large | Integrated | 20.0 | 0.7 | 4.3 | 15.0 | 328 |
| 21 | Medium | Integrated | 19.0 | 0.0 | 4.4 | 16.3 | 252 |
| 22 | Hospital-based | Non-Integrated | 10.9 | 0.7 | 2.8 | 7.6 | 605 |
| 23 | Small | Integrated | 8.8 | 0.0 | 2.0 | 6.8 | 66 |
| 24 | Hospital-based | Non-Integrated | 18.0 | 1.0 | 3.3 | 14.1 | 547 |

^a^Small Clinic – 0-2,500 clients; Medium Clinic – 2,500-10,000 clients; Large Clinic - >10,000 clients; Hospital-based clinic – Outpatient clinic based at facility that also provided inpatient hospital services

^b^ Non-integrated - ART services provided during standalone clinic session; Integrated – ART services integrated with other primary care services

^c^ Number of clients in a facility clinic population over number of providers offering ART services at the at facility, averaged quarterly

### Table S2: Description of RIAS Categories and Abbreviations

| **Standard RIAS Codes** | |
| --- | --- |
| **Functional Group** | **Provider or Client Communication Behaviors (i.e., statement types)** |
| Data Gathering | Medical Questions (Open- or Closed-Ended):  -Individual & family history;  -Therapeutic interventions (tests & treatments)  -Question format closed and open noted) |
|  | Psychosocial Questions: (Open- or Closed-Ended):  -Emotional reactions and coping, social relationships  -Lifestyle and self-care |
| Client Education and Counseling | Medical Information-Giving-Medical condition  -Testing and treatment |
|  | Psychosocial Information-Giving  -Emotional reactions and coping, social relationships;   -Lifestyle habits, preventive, self-care behaviors, work issues, finances |
| Building a Relationship | Positive talk: (1) approval; (2) agreement; (3) jokes |
|  | Disagreements and criticisms |
|  | Social talk: non-medical chit-chat |
|  | Emotional talk: (1) concerns; (2) reassurance; (3) empathy, (4) partnering, (5) self-disclosure |
|  | Facilitation: (1) asking for client opinion; (2) asking for understanding; (3) paraphrase; (4) back-channels |
| Process | Orientation: (1) logistics, directions, and instructions; (2) transition statements and fragments |
| **Study-Specific Codes for Person-centrred Communication Behaviors** | |
| Rapport Building | Instances where provider attempts to build rapport and make client more comfortable (e.g., greets, uses client’s name, laughs, show approval approves, expresses concern) |
| PCC Micropractice | Instances where provider demonstrates use of brief person-centred communication practices e.g., asking for feedback, providing encouragement, explaining decision rationale). |
| Assessing Barriers to Care | Instances where provider seeks to assess or gather information about the hinderances or challenges a client is facing with accessing HIV Care |
| Person-centred Counseling | Instances where provider counseling incorporate person-centred principles (e.g., empathy, offering encouragement, or asking for understanding) |
| Shared decision-making | Instances where provider invites client to partner participate in making decisions about care plan that are mutually agreeable (e.g., when to schedule next appointment, coordinating lab testing and pharmacy refills). |
| Discretionary Power | Instances when HIV provider uses their discretion to better accommodate client needs and preferences, even if it is outside the norms of practice |

### Table S3. Client-Provider Communication across Characteristics using RIAS (n=478)

|  | Partnership (Doc), mean (SD) | Medical question (Doc), mean (SD) | Psychosocial question (Doc), mean (SD) | Medical Information (Doc), mean (SD) | Psychosocial information (Doc), mean (SD) | Psychosocial-Medical Ratio, mean (SD) | Provider Speech Ratio, mean (SD) | Open-Ended question (Doc), mean (SD) | Rapport building, n (%) | PCC micropractice, n (%) | Barriers to HIV care, n (%) | PCC Counseling, n (%) | Shared decision-making, n (%) | Discretionary Power, n (%) | Interaction length, min, median (IQR) |
| --- | --- | --- | --- | --- | --- | --- | --- | --- | --- | --- | --- | --- | --- | --- | --- |
| **Patient** |  |  |  |  |  |  |  |  |  |  |  |  |  |  |  |
| Sex, |  |  |  |  |  |  |  |  |  |  |  |  |  |  |  |
| Female | 0.23 (0.11) | 0.20 (0.11) | 0.06 (0.05) | 0.28 (0.17) | 0.04 (0.06) | 0.17 (0.14) | 0.59 (0.07) | 0.02 (0.04) | 266 (33.1) | 197 (24.5) | 116 (14.4) | 117 (14.6) | 79 (9.8) | 28 (3.5) | 7.4 (5.2-11.9) |
| Male | 0.26 (0.12) | 0.19 (0.10) | 0.04 (0.04) | 0.29 (0.16) | 0.04 (0.06) | 0.15 (0.13) | 0.59 (0.07) | 0.02 (0.03) | 159 (32.1) | 123 (24.8) | 80 (16.2) | 81 (16.4) | 35 (7.1) | 17 (3.4) | 8.2 (5.8-12.0) |
|  |  |  |  |  |  |  |  |  |  |  |  |  |  |  |  |
| Age |  |  |  |  |  |  |  |  |  |  |  |  |  |  |  |
| 18 – 30  years | 0.22 (0.10) | 0.19 (0.12) | 0.05 (0.05) | 0.33 (0.18) | 0.04 (0.06) | 0.16 (0.14) | 0.61 (0.06) | 0.03 (0.04) | 79 (30.7) | 64 (24.9) | 40 (15.6) | 43 (16.7) | 23 (8.9) | 8  (3.1) | 7.5 (5.5-11.4) |
| 31 – 40  years | 0.25 (0.12) | 0.19 (0.10) | 0.06 (0.05) | 0.29 (0.17) | 0.05 (0.07) | 0.18 (0.15) | 0.58 (0.07) | 0.02 (0.04) | 141 (31.3) | 109 (24.2) | 72 (16.0) | 75 (16.6) | 38 (8.4) | 16 (3.5) | 8.4 (5.7-12.3) |
| 41 – 50  years | 0.26 (0.12) | 0.21 (0.12) | 0.05 (0.05) | 0.26 (0.13) | 0.04 (0.05) | 0.17 (0.13) | 0.57 (0.07) | 0.02 (0.03) | 122 (33.4) | 93 (25.5) | 52 (14.2) | 52 (14.2) | 33 (9.0) | 13 (3.6) | 7.8 (5.4-12.0) |
| >50 years | 0.24 (0.12) | 0.19 (0.10) | 0.04 (0.05) | 0.33 (0.17) | 0.02 (0.04) | 0.12 (0.10) | 0.59 (0.07) | 0.02 (0.05) | 57 (35.2) | 42 (25.9) | 21 (13.0) | 26 (16.0) | 10 (6.2) | 6  (3.7) | 7.1 (5.0-9.2) |
|  |  |  |  |  |  |  |  |  |  |  |  |  |  |  |  |
| Marital Status |  |  |  |  |  |  |  |  |  |  |  |  |  |  |  |
| Single | 0.24 (0.11) | 0.18 (0.10) | 0.06 (0.05) | 0.29 (0.17) | 0.04 (0.06) | 0.18 (0.14) | 0.59 (0.08) | 0.03 (0.05) | 56 (30.4) | 51 (27.7) | 29 (15.8) | 31 (16.8) | 14 (7.6) | 3  (1.6) | 7.3 (5.1-11.5) |
| Married | 0.25 (0.12) | 0.20 (0.11) | 0.05 (0.05) | 0.29 (0.16) | 0.04 (0.06) | 0.16 (0.14) | 0.59 (0.06) | 0.02 (0.04) | 225 (33.7) | 160 (24.0) | 94 (14.1) | 106 (15.9) | 60 (9.0) | 22 (3.3) | 7.7 (5.5-11.3) |
| Divorced | 0.23 (0.10) | 0.19 (0.12) | 0.05 (0.04) | 0.29 (0.17) | 0.06 (0.08) | 0.20 (0.15) | 0.59 (0.07) | 0.02 (0.04) | 44 (30.1) | 35 (24.0) | 22 (15.1) | 28 (19.2) | 8  (5.5) | 9  (6.2) | 8.6 (5.8-14.9) |
| Widowed | 0.21 (0.12) | 0.21 (0.12) | 0.05 (0.03) | 0.30 (0.16) | 0.04 (0.05) | 0.15 (0.12) | 0.58 (0.06) | 0.01 (0.02) | 30 (32.3) | 24 (25.8) | 15 (16.1) | 10 (10.8) | 10 (10.8) | 4  (4.3) | 7.8 (5.8-11.2) |
|  |  |  |  |  |  |  |  |  |  |  |  |  |  |  |  |
| Education |  |  |  |  |  |  |  |  |  |  |  |  |  |  |  |
| None | 0.18 (0.09) | 0.24 (0.15) | 0.05 (0.06) | 0.31 (0.19) | 0.02 (0.04) | 0.12 (0.11) | 0.58 (0.09) | 0.01 (0.02) | 17 (37.8) | 9 (20.0) | 4  (8.9) | 8 (17.8) | 5 (11.1) | 2  (4.4) | 5.1 (4.6-12.5) |
| Primary | 0.24 (0.12) | 0.20 (0.10) | 0.06 (0.05) | 0.27 (0.15) | 0.04 (0.06) | 0.18 (0.14) | 0.57 (0.06) | 0.03 (0.05) | 103 (33.2) | 77 (24.8) | 44 (14.2) | 44 (14.2) | 29 (9.4) | 13 (4.2) | 7.5 (5.6-11.3) |
| Secondary | 0.25 (0.11) | 0.19 (0.11) | 0.06 (0.05) | 0.30 (0.17) | 0.05 (0.06) | 0.17 (0.14) | 0.59 (0.07) | 0.02 (0.04) | 207 (30.9) | 166 (24.8) | 107 (16.0) | 114 (17.0) | 55 (8.2) | 21 (3.1) | 7.9 (5.8-11.9) |
| University | 0.24 (0.14) | 0.17 (0.11) | 0.02 (0.02) | 0.34 (0.20) | 0.05 (0.07) | 0.13 (0.14) | 0.61 (0.09) | 0.03 (0.05) | 21 (30.9) | 19 (27.9) | 10 (14.7) | 13 (19.1) | 3  (4.4) | 2 (2.9) | 7.3 (4.5-12.7) |
|  |  |  |  |  |  |  |  |  |  |  |  |  |  |  |  |
| Time since enrollment in care |  |  |  |  |  |  |  |  |  |  |  |  |  |  |  |
| <6 months | 0.24 (0.11) | 0.17 (0.11) | 0.05 (0.06) | 0.28 (0.17) | 0.05 (0.06) | 0.19 (0.15) | 0.60 (0.08) | 0.03 (0.04) | 36 (33.6) | 29 (27.1) | 12 (11.2) | 19 (17.8) | 7  (6.5) | 4  (3.7) | 7.0 (5.2-11.0) |
| 6 months –  1 year | 0.23 (0.12) | 0.21 (0.14) | 0.04 (0.05) | 0.30 (0.20) | 0.04 (0.05) | 0.14 (0.14) | 0.59 (0.08) | 0.02 (0.03) | 37 (31.9) | 29 (25) | 20 (17.2) | 17 (14.7) | 8  (6.9) | 5  (4.3) | 7.9 (4.7-12.8) |
| 1 – 2 years | 0.23 (0.12) | 0.19 (0.09) | 0.05 (0.04) | 0.28 (0.17) | 0.03 (0.05) | 0.15 (0.13) | 0.59 (0.05) | 0.02 (0.03) | 46 (35.4) | 32 (24.6) | 22 (16.9) | 19 (14.6) | 9  (6.9) | 2  (1.5) | 7.7 (5.6-11.1) |
| 2 – 5 years | 0.24 (0.11) | 0.19 (0.11) | 0.06 (0.05) | 0.31 (0.17) | 0.04 (0.06) | 0.17 (0.14) | 0.59 (0.07) | 0.02 (0.03) | 102 (31.9) | 83 (25.9) | 51 (15.9) | 48 (15) | 30 (9.4) | 6  (1.9) | 8.0 (6.4-12.5) |
| > 5 years | 0.26 (0.12) | 0.20 (0.11) | 0.06 (0.05) | 0.28 (0.15) | 0.04 (0.06) | 0.17 (0.14) | 0.58 (0.07) | 0.03 (0.04) | 176 (31.6) | 133 (23.9) | 80 (14.4) | 92 (16.5) | 50  (9.0) | 26 (4.7) | 7.8 (5.7-11.9) |
|  |  |  |  |  |  |  |  |  |  |  |  |  |  |  |  |
| Enrollment WHO stage |  |  |  |  |  |  |  |  |  |  |  |  |  |  |  |
| WHO  Stage 1 | 0.24 (0.11) | 0.20 (0.11) | 0.05 (0.04) | 0.28 (0.17) | 0.04 (0.06) | 0.15 (0.14) | 0.58 (0.07) | 0.02 (0.03) | 175 (33) | 131 (24.7) | 83 (15.7) | 79 (14.9) | 48 (9.1) | 14 (2.6) | 8.0 (5.2-11.8) |
| WHO  Stage 2 | 0.23 (0.11) | 0.17 (0.09) | 0.05 (0.04) | 0.32 (0.19) | 0.03 (0.05) | 0.14 (0.12) | 0.59 (0.07) | 0.01 (0.03) | 49 (32.9) | 33 (22.1) | 23 (15.4) | 24 (16.1) | 15 (10.1) | 5 (3.4) | 8.0 (5.2-10.0) |
| WHO  Stage 3 | 0.26 (0.13) | 0.21 (0.10) | 0.06 (0.05) | 0.26 (0.15) | 0.05 (0.07) | 0.18 (0.15) | 0.58 (0.06) | 0.02 (0.03) | 38 (33.6) | 25 (22.1) | 18 (15.9) | 17 (15) | 9  (8) | 6  (5.3) | 7.9 (4.6-13.6) |
| WHO  Stage 4 | 0.31 (0.16) | 0.14 (0.08) | 0.03 (0.01) | 0.24 (0.06) | 0.07 (0.05) | 0.20 (0.12) | 0.66 (0.07) | 0.02 (0.03) | 2  (25.0) | 2 (25.0) | 2 (25.0) | 1 (12.5) | (0) | 1 (12.5) | 7.5 (5.2-10.1) |
|  |  |  |  |  |  |  |  |  |  |  |  |  |  |  |  |
| **Provider** |  |  |  |  |  |  |  |  |  |  |  |  |  |  |  |
| Sex |  |  |  |  |  |  |  |  |  |  |  |  |  |  |  |
| Female | 0.25 (0.12) | 0.19 (0.10) | 0.05 (0.05) | 0.29 (0.17) | 0.04 (0.06) | 0.17 (0.14) | 0.59 (0.07) | 0.02 (0.04) | 209 (31.9) | 163 (24.9) | 92 (14) | 105 (16) | 60 (9.2) | 26  (4.0) | 7.9 (5.6-12.0) |
| Male | 0.24 (0.12) | 0.20 (0.12) | 0.05 (0.05) | 0.29 (0.16) | 0.04 (0.06) | 0.16 (0.14) | 0.58 (0.07) | 0.02 (0.04) | 216 (33.6) | 157 (24.4) | 104 (16.2) | 93 (14.5) | 54 (8.4) | 19 (3.0) | 7.7 (5.2-11.9) |
|  |  |  |  |  |  |  |  |  |  |  |  |  |  |  |  |
| Provider Type |  |  |  |  |  |  |  |  |  |  |  |  |  |  |  |
| Nurse | 0.23 (0.11) | 0.15 (0.09) | 0.05 (0.04) | 0.32 (0.18) | 0.06 (0.08) | 0.18 (0.16) | 0.59 (0.07) | 0.01 (0.02) | 61 (31.6) | 52 (26.9) | 22 (11.4) | 33 (17.1) | 16 (8.3) | 9  (4.7) | 12.2 (7.0-18.1) |
| Clinical  Officer | 0.25 (0.12) | 0.21 (0.11) | 0.05 (0.05) | 0.28 (0.16) | 0.04 (0.05) | 0.15 (0.13) | 0.59 (0.07) | 0.03 (0.04) | 313 (32.8) | 234 (24.6) | 144 (15.1) | 146 (15.3) | 86 (9) | 30 (3.1) | 7.4 (5.2-11.0) |
| Medical  Officer | 0.21 (0.11) | 0.18 (0.09) | 0.06 (0.05) | 0.29 (0.19) | 0.05 (0.06) | 0.20 (0.14) | 0.59 (0.07) | 0.01 (0.02) | 51 (33.6) | 34 (22.4) | 30 (19.7) | 19 (12.5) | 12 (7.9) | 6  (3.9) | 8.5 (5.8-12.1) |
|  |  |  |  |  |  |  |  |  |  |  |  |  |  |  |  |
| **Interaction** |  |  |  |  |  |  |  |  |  |  |  |  |  |  |  |
| Interaction language |  |  |  |  |  |  |  |  |  |  |  |  |  |  |  |
| Nyanja | 0.25 (0.12) | 0.20 (0.11) | 0.05 (0.05) | 0.28 (0.16) | 0.04 (0.06) | 0.17 (0.14) | 0.58 (0.07) | 0.03 (0.04) | 227 (32.9) | 172 (24.9) | 93 (13.5) | 107 (15.5) | 64 (9.3) | 28 (4.1) | 7.5 (5.7-11.7) |
| English | 0.23 (0.11) | 0.19 (0.11) | 0.05 (0.04) | 0.31 (0.18) | 0.04 (0.05) | 0.16 (0.13) | 0.60 (0.06) | 0.01 (0.03) | 132 (31.4) | 102 (24.2) | 71 (16.9) | 69 (16.4) | 33 (7.8) | 14 (3.3) | 8.3 (5.3 -13.3) |
| Bemba | 0.23 (0.13) | 0.20 (0.10) | 0.05 (0.05) | 0.28 (0.15) | 0.04 (0.06) | 0.16 (0.13) | 0.58 (0.07) | 0.02 (0.03) | 66 (35.5) | 46 (24.7) | 32 (17.2) | 22 (11.8) | 17 (9.1) | 3  (1.6) | 7.3 (4.7-10.1) |
|  |  |  |  |  |  |  |  |  |  |  |  |  |  |  |  |
| Sex Concordance (Patient – Provider) |  |  |  |  |  |  |  |  |  |  |  |  |  |  |  |
| Female -  Female | 0.24 (0.12) | 0.20 (0.10) | 0.06 (0.05) | 0.28 (0.16) | 0.04 (0.06) | 0.17 (0.14) | 0.59 (0.07) | 0.02 (0.04) | 141 (32.4) | 111 (25.5) | 56 (12.9) | 66 (15.2) | 40 (9.2) | 21 (4.8) | 7.5 (5.1-11.9) |
| Female –  Male | 0.22 (0.11) | 0.21 (0.12) | 0.06 (0.06) | 0.28 (0.17) | 0.04 (0.06) | 0.17 (0.14) | 0.59 (0.06) | 0.02 (0.04) | 125 (34) | 86 (23.4) | 60 (16.3) | 51 (13.9) | 39 (10.6) | 7  (1.9) | 7.32 (5.5 - 11.7) |
| Male –  Female | 0.25 (0.12) | 0.18 (0.10) | 0.04 (0.04) | 0.30 (0.17) | 0.05 (0.06) | 0.17 (0.14) | 0.59 (0.07) | 0.02 (0.03) | 68 (30.9) | 52 (23.6) | 36 (16.4) | 39 (17.7) | 20 (9.1) | 5  (2.3) | 8.3 (6.1 -12.2) |
| Male –  Male | 0.26 (0.12) | 0.19 (0.10) | 0.04 (0.04) | 0.29 (0.16) | 0.03 (0.06) | 0.14 (0.13) | 0.58 (0.08) | 0.02 (0.04) | 91 (33.1) | 71 (25.8) | 44 (16) | 42 (15.3) | 15 (5.5) | 12 (4.4) | 8.0 (5.2-11.9) |
|  |  |  |  |  |  |  |  |  |  |  |  |  |  |  |  |
| Time Period, n (%) |  |  |  |  |  |  |  |  |  |  |  |  |  |  |  |
| 01 Aug  2019 –  31 Mar  2020 | 0.15 (0.06) | 0.15 (0.08) | 0.03 (0.03) | 0.16 (0.08) | 0.03 (0.04) | 0.16 (0.15) | 0.57 (0.05) | 0.00 (0.01) | 68 (38.4) | 31 (17.5) | 33 (18.6) | 11 (6.2) | 26 (14.7) | 8  (4.5) | 8.6 (6.3 -12.0) |
| 01 Apr  2020 –  30 Aug  2020 | 0.22 (0.11) | 0.20 (0.11) | 0.05 (0.05) | 0.28 (0.18) | 0.03 (0.04) | 0.15 (0.13) | 0.57 (0.07) | 0.01 (0.01) | 50 (34.5) | 32 (22.1) | 22 (15.2) | 15 (10.3) | 22 (15.2) | 4  (2.8) | 7.1 (5.8 -11.3) |
| 01 Sept  2020 –  30 Nov  2021 | 0.27 (0.12) | 0.21 (0.11) | 0.06 (0.05) | 0.32 (0.16) | 0.05 (0.06) | 0.16 (0.14) | 0.59 (0.07) | 0.03 (0.04) | 307 (31.5) | 257 (26.3) | 141 (14.4) | 172 (17.6) | 66 (6.8) | 33 (3.4) | 7.7 (5.0 -12.0) |
|  |  |  |  |  |  |  |  |  |  |  |  |  |  |  |  |
| Interaction Length |  |  |  |  |  |  |  |  |  |  |  |  |  |  |  |
| <5 minutes | 0.26 (0.13) | 0.24 (0.14) | 0.05 (0.06) | 0.25 (0.18) | 0.02 (0.05) | 0.13 (0.14) | 0.58 (0.08) | 0.03 (0.05) | 85 (39.5) | 59 (27.4) | 25 (11.6) | 29 (13.5) | 13  (6.0) | 4  (1.9) | 3.8 (3.1 -4.4) |
| 5 to 10  minutes | 0.24 (0.11) | 0.19 (0.10) | 0.05 (0.05) | 0.29 (0.16) | 0.03 (0.05) | 0.15 (0.13) | 0.59 (0.06) | 0.02 (0.04) | 196 (34.3) | 149 (26) | 80 (14) | 87 (15.2) | 46  (8.0) | 14 (2.4) | 7.1 (6.1-8.2) |
| >10 minutes | 0.23 (0.11) | 0.17 (0.10) | 0.05 (0.04) | 0.31 (0.16) | 0.06 (0.07) | 0.19 (0.14) | 0.59 (0.07) | 0.02 (0.03) | 144 (28.2) | 112 (21.9) | 91 (17.8) | 82 (16) | 55 (10.8) | 27 (5.3) | 14.0 (11.9 -17.1) |
|  |  |  |  |  |  |  |  |  |  |  |  |  |  |  |  |
| **Facility** |  |  |  |  |  |  |  |  |  |  |  |  |  |  |  |
| Facility Type^a^ |  |  |  |  |  |  |  |  |  |  |  |  |  |  |  |
| Small Clinic | 0.25 (0.11) | 0.18 (0.11) | 0.04 (0.04) | 0.32 (0.16) | 0.04 (0.06) | 0.14 (0.13) | 0.59 (0.07) | 0.02 (0.04) | 87 (31.6) | 65 (23.6) | 38 (13.8) | 42 (15.3) | 32 (11.6) | 11  (4.0) | 9.3 (5.6-14.3) |
| Medium  Clinic | 0.24 (0.12) | 0.22 (0.11) | 0.05 (0.05) | 0.28 (0.16) | 0.03 (0.06) | 0.14 (0.13) | 0.57 (0.06) | 0.02 (0.04) | 163 (34.2) | 113 (23.7) | 75 (15.7) | 72 (15.1) | 43  (9.0) | 11 (2.3) | 7.0 (5.0 - 8.6) |
| Large Clinic | 0.24 (0.12) | 0.20 (0.11) | 0.07 (0.06) | 0.26 (0.18) | 0.05 (0.05) | 0.21 (0.14) | 0.58 (0.08) | 0.03 (0.05) | 71 (35.9) | 48 (24.2) | 34 (17.2) | 31 (15.7) | 11 (5.6) | 3  (1.5) | 7.8 (5.4 -11.8) |
| Hospital-  Based Clinic | 0.24 (0.12) | 0.18 (0.09) | 0.05 (0.04) | 0.30 (0.17) | 0.05 (0.06) | 0.18 (0.15) | 0.61 (0.06) | 0.02 (0.03) | 104 (29.9) | 94 (27) | 49 (14.1) | 53 (15.2) | 28  (8.0) | 20 (5.7) | 9.8 (5.9 - 14.8) |
|  |  |  |  |  |  |  |  |  |  |  |  |  |  |  |  |
| ART Integration^b^ |  |  |  |  |  |  |  |  |  |  |  |  |  |  |  |
| Non-  Integrated | 0.24 (0.11) | 0.18 (0.10) | 0.05 (0.04) | 0.30 (0.17) | 0.04 (0.06) | 0.16 (0.13) | 0.60 (0.07) | 0.02 (0.03) | 278 (31.5) | 222 (25.2) | 131 (14.9) | 138 (15.6) | 77 (8.7) | 36 (4.1) | 8.3 (5.8 -12.9) |
| Integrated | 0.24 (0.13) | 0.22 (0.11) | 0.06 (0.06) | 0.26 (0.15) | 0.03 (0.06) | 0.16 (0.14) | 0.57 (0.07) | 0.03 (0.05) | 147 (35.3) | 98 (23.6) | 65 (15.6) | 60 (14.4) | 37 (8.9) | 9  (2.2) | 6.9 (5.0 -9.6) |
|  |  |  |  |  |  |  |  |  |  |  |  |  |  |  |  |
| Client:Provider Ratio^c^ |  |  |  |  |  |  |  |  |  |  |  |  |  |  |  |
| <250 clients  per provider | 0.25 (0.11) | 0.19 (0.11) | 0.05 (0.05) | 0.29 (0.15) | 0.04 (0.06) | 0.15 (0.14) | 0.59 (0.07) | 0.02 (0.04) | 146 (33.9) | 106 (24.6) | 58 (13.5) | 58 (13.5) | 44 (10.2) | 19 (4.4) | 8.4 (5.7 -13.0) |
| 250-500  clients per  provider | 0.24 (0.12) | 0.19 (0.11) | 0.06 (0.05) | 0.30 (0.18) | 0.04 (0.06) | 0.18 (0.14) | 0.59 (0.08) | 0.02 (0.04) | 135 (32.5) | 100 (24) | 70 (16.8) | 60 (14.4) | 39 (9.4) | 12 (2.9) | 6.9 (4.8-10.8) |
| >500 clients  per provider | 0.24 (0.12) | 0.21 (0.11) | 0.05 (0.05) | 0.27 (0.16) | 0.04 (0.06) | 0.16 (0.14) | 0.58 (0.06) | 0.02 (0.03) | 144 (31.9) | 114 (25.3) | 68 (15.1) | 80 (17.7) | 31 (6.9) | 14 (3.1) | 7.9 (6.1 -12.0) |

^a^Small Clinic – 0-2,500 clients; Medium Clinic – 2,500-10,000 clients; Large Clinic - >10,000 clients; Hospital-based clinic – Outpatient clinic based at facility that also provided inpatient hospital services

^b^ Non-integrated - ART services provided during standalone clinic session; Integrated – ART services integrated with other primary care services

^c^Number of clients in a facility clinic population over number of providers offering ART services at the at facility, averaged quarterly

### Table S4. Goodness-of-Fit Statistics for Latent Class Models with Different Number of Classes

| **Model** | **N** | **Log-Likelihood** | **DF** | **AIC** | **BIC** | **Entropy** |
| --- | --- | --- | --- | --- | --- | --- |
| 1-Class | 478 | 2581.789 | 22 | -5119.579 | -5027.847 | 1 |
| 2-Class | 478 | 2849.066 | 37 | -5624.131 | -5469.856 | 0.877 |
| 3-Class | 478 | 3018.69 | 52 | -5933.381 | -5716.561 | 0.844 |
| 4-Class | 478 | 3213.356 | 67 | -6292.713 | -6013.349 | 0.866 |
| 5-Class | 478 | 3338.236 | 82 | -6512.473 | -6170.565 | 0.866 |
| 6-Class | 478 | 3405.892 | 97 | -6617.783 | -6213.331 | 0.868 |

AIC, Akaike Information Criteria; BIC, Bayesian Information Criteria; DF, Degrees of Freedom. Lower values for AIC and BIC indicate better fit. Entropy values closer to 1 indicate stronger separate between classes.

### Table S5: Item Response Probabilities for Latent Classes (n=478)

|  | **Medically-Oriented Interaction, Minimal PCC Behaviors**  **(n = 237)** | **Balanced Medical/**  **Nonmedical Interaction**  **(n = 95)** | **Medically-Oriented Interaction, Good PCC Behaviors**  **(n = 111)** | **Highly Person-centred Interaction**  **(n = 35)** |
| --- | --- | --- | --- | --- |
| Partnership (Doc) | 0.252 | 0.277 | 0.189 | 0.238 |
| Medical question (Doc) | 0.238 | 0.180 | 0.152 | 0.112 |
| Psychosocial question (Doc) | 0.032 | 0.118 | 0.034 | 0.050 |
| Medical Information (Doc) | 0.217 | 0.198 | 0.505 | 0.283 |
| Psychosocial information (Doc) | 0.013 | 0.053 | 0.030 | 0.203 |
| Psychosocial-Medical Ratio | 0.092 | 0.317 | 0.090 | 0.401 |
| Provider Speech Ratio | 0.565 | 0.564 | 0.644 | 0.611 |
| Open-Ended question (Doc) | 0.023 | 0.030 | 0.016 | 0.019 |
| Rapport building | 0.888 | 0.903 | 0.907 | 0.799 |
| Small PCC practice | 0.565 | 0.606 | 0.871 | 0.870 |
| Barriers to HIV care | 0.348 | 0.360 | 0.516 | 0.605 |
| Person-centred Counseling | 0.273 | 0.298 | 0.694 | 0.746 |
| Shared decision-making | 0.207 | 0.228 | 0.279 | 0.335 |
| Discretionary Power | 0.065 | 0.088 | 0.105 | 0.259 |
| Interaction length, min, median (IQR) | 7.0 (4.9 – 10.1) | 7.48 (5.8 – 13.6) | 9.4 (6.7 – 12.7) | 11.2 (7.1 – 17.8) |

### Table S6. Predictive Margins of Latent Class Distribution Across Client, Provider, Interaction, and Facility Characteristics from Multinomial Logistic Regression (n=478)

|  | **Medically-Oriented Interaction, Minimal PCC Behaviors** | **Balanced Medical/**  **Nonmedical Interaction, Low PCC Behaviors** | **Medically-Oriented Interaction, Good PCC Behaviors** | **Highly Person-centred Interaction** |
| --- | --- | --- | --- | --- |
| **Client** |  |  |  |  |
| Sex |  |  |  |  |
| Female | 50.3%  (45.0-55.6%) | 21.8%  (17.2-26.4%) | 22.2%  (17.7-26.7%) | 5.7%  (3.2-8.1%) |
| Male | 48.4%  (41.3-55.5%) | 16.6%  (11.0-22.2%) | 24.7%  (18.3-31.0%) | 10.4%  (5.8-14.9%) |
|  |  |  |  |  |
| Age |  |  |  |  |
| 18 - 30 years | 44.3%  (33.0-55.6%) | 17.4%  (8.5-26.2%) | 30.5%  (20.5-40.5%) | 7.8%  (1.3-14.4%) |
| 31 - 40 years | 46.1%  (39.1-53.2%) | 20.0%  (14.1-25.9%) | 22.7%  (16.8-28.6%) | 11.2%  (6.1-16.3%) |
| 41 - 50 years | 54.6%  (46.8-62.3%) | 23.0%  (16.1-29.9%) | 15.6%  (9.7-21.6%) | 6.8%  (2.7-10.8%) |
| >50 years | 52.6%  (39.0-66.2%) | 15.7%  (5.0-26.4%) | 30.5%  (16.6-44.3%) | 1.3%  (0.0-4.0%) |
|  |  |  |  |  |
| Marital Status |  |  |  |  |
| Single | 49.8%  (37.9-61.7%) | 22.6%  (12.2-33.0%) | 20.5%  (12.3-28.6%) | 7.1%  (0.8-13.5%) |
| Married | 49.9%  (44.4-55.5%) | 20.0%  (15.2-24.8%) | 23.8%  (19.0-28.6%) | 6.2%  (3.6-8.9%) |
| Divorce | 45.2%  (31.7-58.6%) | 25.0%  (13.0-37.0%) | 18.8%  (8.3-29.2%) | 11.1%  (4.1-18.0%) |
| Widowed | 49.2%  (32.1-66.3%) | 8.3%  (0.0-16.8%) | 29.9%  (14.6-45.3%) | 12.6%  (0.0-30.3%) |
|  |  |  |  |  |
| Education |  |  |  |  |
| None | 51.7%  (28.6-74.8%) | 6.2%  (0.0-17.8%) | 33.3%  (9.4-57.1%) | 8.8%  (0.0-25.4%) |
| Primary | 50.9%  (42.6-59.2%) | 24.0%  (16.4-31.5%) | 17.5%  (11.0-24.0%) | 7.6%  (3.0-12.2%) |
| Secondary | 47.7%  (41.7-53.6%) | 21.1%  (16.1-26.1%) | 24.3%  (19.5-29.0%) | 7.0%  (4.1-9.8%) |
| University | 55.6%  (36.0-75.3%) | 5.2%  (0.0-14.0%) | 29.3%  (11.8-46.8%) | 9.9%  (0.0-21.5%) |
|  |  |  |  |  |
| Time since enrollment in care |  |  |  |  |
| <6 months | 45.7%  (29.7-61.8%) | 21.1%  (8.1-34.2%) | 20.7%  (9.7-31.8%) | 12.4%  (1.5-23.3%) |
| 6 months – 1 year | 52.3%  (38.5-66.1%) | 16.4%  (6.6-26.3%) | 22.1%  (11.5-32.7%) | 9.2%  (0.9-17.6%) |
| 1 – 2 years | 52.2%  (40.3-64.0%) | 20.5%  (10.1-31.0%) | 22.5%  (12.6-32.5%) | 4.8%  (0.0-10.6%) |
| 2 – 5 years | 47.7%  (39.0-56.4%) | 17.5%  (10.8-24.2%) | 26.1%  (19.0-33.2%) | 8.7%  (3.5-13.9%) |
| >5 years | 49.9%  (42.9-56.9%) | 21.9%  (15.1-28.7%) | 22.2%  (16.1-28.3%) | 6.0%  (2.8-9.1%) |
|  |  |  |  |  |
| Enrollment WHO stage |  |  |  |  |
| WHO Stage 1 | 47.5%  (41.5-53.6%) | 21.6%  (16.2-27.0%) | 25.6%  (20.5-30.7%) | 5.3%  (2.7-7.9%) |
| WHO Stage 2 | 48.3%  (36.5-60.1%) | 16.4%  (6.6-26.2%) | 23.0%  (12.5-33.5%) | 12.3%  (3.6-21.0%) |
| WHO Stage 3 | 57.1%  (42.8-71.4%) | 17.2%  (6.9-27.4%) | 13.3%  (3.7-22.8%) | 12.5%  (1.2-23.8%) |
| WHO Stage 4 | 64.3%  (23.2-105.4%) | 9.4%  (0.0-39.4%) | 0.4%  (0.0-5.2%) | 25.9%  (-12.2-64.1%) |
|  |  |  |  |  |
| **Provider** | | | | |
| Sex |  |  |  |  |
| Female | 44.5%  (38.0-50.9%) | 23.9%  (18.0-29.8%) | 23.7%  (18.6-28.8%) | 7.9%  (4.6-11.3%) |
| Male | 54.3%  (48.2-60.3%) | 16.4%  (11.7-21.1%) | 22.6%  (17.3-27.9%) | 6.8%  (3.6-9.9%) |
|  |  |  |  |  |
| Provider Type |  |  |  |  |
| Nurse | 42.3%  (30.5-54.0%) | 16.7%  (7.6-25.7%) | 31.8%  (20.3-43.2%) | 9.3%  (2.0-16.6%) |
| Clinical Officer | 53.4%  (48.5-58.2%) | 19.0%  (15.0-23.1%) | 21.7%  (17.5-25.9%) | 5.9%  (3.7-8.2%) |
| Medical Officer | 32.6%  (20.1-45.2%) | 28.7%  (16.4-41.0%) | 21.5%  (11.6-31.3%) | 17.2%  (5.5-28.8%) |
|  |  |  |  |  |
| **Interaction** | | | | |
| Interaction language |  |  |  |  |
| Nyanja | 49.7%  (43.9-55.5%) | 19.5%  (14.6-24.3%) | 20.7%  (15.8-25.5%) | 10.2%  (6.5-13.9%) |
| English | 48.6%  (40.6-56.6%) | 20.2%  (13.8-26.6%) | 27.5%  (20.7-34.2%) | 3.7%  (1.1-6.4%) |
| Bemba | 50.2%  (39.7-60.6%) | 20.1%  (10.5-29.7%) | 20.5%  (11.2-29.8%) | 9.2%  (1.0-17.4%) |
|  |  |  |  |  |
| Client-Provider Sex Concordance |  |  |  |  |
| No | 53.2%  (47.9-58.6%) | 17.0%  (12.6-21.5%) | 22.7%  (18.0-27.3%) | 7.1%  (4.2-10.0%) |
| Yes | 45.1%  (38.6-51.6%) | 23.5%  (17.7-29.3%) | 23.8%  (18.4-29.2%) | 7.6%  (4.3-10.8%) |
|  |  |  |  |  |
| Time Period |  |  |  |  |
| 01 Aug 2019 – 31 Mar 2020 | 78.3%  (69.8-86.8%) | 15.5%  (7.9-23.1%) | 3.1%  (0.0-6.4%) | 3.1%  (0.0-6.4%) |
| 01 Apr 2020 – 30 Aug 2020 | 48.5%  (36.0-60.9%) | 23.8%  (12.6-35.0%) | 24.7%  (13.3-36.0%) | 3.1%  (0.0-7.8%) |
| 01 Sept 2020 – 30 Nov 2021 | 41.8%  (36.8-46.8%) | 20.0%  (15.9-24.2%) | 28.6%  (24.1-33.2%) | 9.5%  (6.4-12.7%) |
|  |  |  |  |  |
| Interaction Length |  |  |  |  |
| <5 minutes | 63.5%  (54.7-72.3%) | 22.1%  (13.9-30.3%) | 11.4%  (6.0-16.9%) | 3.0%  (0.0-6.6%) |
| 5 to 10 minutes | 48.8%  (42.5-55.2%) | 19.0%  (13.7-24.2%) | 26.8%  (20.9-32.7%) | 5.4%  (2.4-8.4%) |
| >10 minutes | 40.7%  (33.0-48.3%) | 20.1%  (13.7-26.5%) | 27.1%  (20.3-34.0%) | 12.1%  (7.3-16.9%) |
|  |  |  |  |  |
| **Facility** |  |  |  |  |
| Facility Type^a^ |  |  |  |  |
| Small Clinic | 44.5%  (32.7-56.3%) | 16.5%  (6.0-26.9%) | 25.9%  (15.5-36.2%) | 13.2%  (3.0-23.3%) |
| Medium Clinic | 57.0%  (50.1-63.9%) | 14.6%  (9.6-19.7%) | 23.5%  (17.4-29.6%) | 4.9%  (1.5-8.2%) |
| Large Clinic | 45.4%  (32.6-58.2%) | 32.8%  (20.2-45.4%) | 19.1%  (9.8-28.4%) | 2.7%  (0.0-5.3%) |
| Hospital-Based Clinic | 42.1%  (32.2-52.0%) | 21.8%  (13.5-30.1%) | 21.3%  (13.7-28.9%) | 14.8%  (7.2-22.5%) |
|  |  |  |  |  |
| ART Integration^b^ |  |  |  |  |
| Non-Integrated | 48.4%  (42.9-54.0%) | 19.4%  (14.9-23.9%) | 26.1%  (21.4-30.9%) | 26.1%  (21.4-30.9%) |
| Integrated | 49.9%  (41.8-57.9%) | 19.8%  (12.8-26.8%) | 16.4%  (10.6-22.3%) | 16.4%  (10.6-22.3%) |
|  |  |  |  |  |
| Client:Provider Ratio^c^ |  |  |  |  |
| <250 clients per provider | 54.6%  (45.7-63.5%) | 21.5%  (13.0-30.0%) | 19.2%  (13.4-25.0%) | 4.7%  (1.5-7.9%) |
| 250-500 clients per provider | 43.6%  (35.7-51.6%) | 14.7%  (9.6-19.8%) | 29.2%  (21.4-37.0%) | 12.5%  (5.6-19.4%) |
| >500 clients per provider | 48.1%  (40.2-56.0%) | 23.7%  (16.5-31.0%) | 21.2%  (14.3-28.1%) | 7.0%  (2.7-11.3%) |

^a^Small Clinic – 0-2,500 clients; Medium Clinic – 2,500-10,000 clients; Large Clinic - >10,000 clients; Hospital-based clinic – Outpatient clinic based at facility that also provided inpatient hospital services

^b^ Non-integrated - ART services provided during standalone clinic session; Integrated – ART services integrated with other primary care services

^c^Number of clients in a facility clinic population over number of providers offering ART services at the at facility, averaged quarterly

### Table S7. Association between Client-Provider Communication and Being Late to Next Visit by 30 days (n=361)

|  | Unadjusted  Risk Ratio  (95% CI) | p-value | Adjusted  Risk Ratio  (95% CI) | p-value |
| --- | --- | --- | --- | --- |
| **Communication Profile** |  |  |  |  |
| Medically Oriented Interaction,  Minimal PCC Behaviors | 1 (REF) | 0.824 | 1 (REF) | 0.824 |
| Balanced Medical/Nonmedical  Interaction | 1.17 (0.70 – 1.98) |  | 1.00 (0.57 – 1.77) |  |
| Medically Oriented Interaction,  Good PCC Behaviors | 1.25 (0.77 – 2.03) |  | 1.24 (0.71 – 2.20) |  |
| Highly Person-centred  Interaction | 1.11 (0.51 – 2.41) |  | 1.31 (0.67 – 2.55) |  |
|  |  |  |  |  |
| **Client** |  |  |  |  |
| Client Sex |  |  |  |  |
| Female | 1 (REF) | 0.422 | 1 (REF) | 0.692 |
| Male | 0.84 (0.55 – 1.28) |  | 0.91 (0.58 – 1.44) |  |
|  |  |  |  |  |
| Age Category |  |  |  |  |
| 18 - 30 years | 1 (REF) | 0.858 | 1 (REF) | 0.953 |
| 31 - 40 years | 0.95 (0.52 – 1.73) |  | 0.95 (0.54 – 1.65) |  |
| 41 - 50 years | 1.13 (0.64 – 2.01) |  | 1.12 (0.55 – 2.27) |  |
| >50 years | 1.2 (0.6 – 2.40) |  | 1.20 (0.46 – 3.14) |  |
|  |  |  |  |  |
| Marital Status |  |  |  |  |
| Single | 1 (REF) | 0.475 | 1 (REF) | 0.328 |
| Married | 0.68 (0.38 – 1.24) |  | 0.52 (0.26 – 1.04) |  |
| Divorced | 1.02 (0.48 – 2.19) |  | 0.67 (0.33 -1.36) |  |
| Widowed | 0.93 (0.39 – 2.21) |  | 0.66 (0.26 – 1.66) |  |
|  |  |  |  |  |
| Education |  |  |  |  |
| None | 1 (REF) | 0.367 | 1 (REF) | 0.376 |
| Primary | 0.55 (0.23 – 1.33) |  | 0.51 (0.17 – 1.54) |  |
| Secondary | 0.57 (0.25 – 1.32) |  | 0.47 (0.17 – 1.33) |  |
| University | 0.27 (0.60 – 1.27) |  | 0.28 (0.06 – 1.20) |  |
|  |  |  |  |  |
| Time since enrollment in care |  |  |  |  |
| <6 months | 1 (REF) | 0.186 | 1 (REF) | 0.055 |
| 6 months – 1 year | 2.0 (0.54 – 7.42) |  | 1.80 (0.35 – 9.16) |  |
| 1 – 2 years | 1.85 (0.50 – 6.88) |  | 1.49 (0.46 – 4.82) |  |
| 2 – 5 years | 3.0 (0.94 – 9.39) |  | 2.46 (0.88 – 6.88) |  |
| >5 years | 3.12 (1.01 – 9.57) |  | 3.15 (1.18 – 8.38) |  |
|  |  |  |  |  |
| Enrollment WHO stage |  |  |  |  |
| WHO Stage 1 | 1 (REF) | 0.044 | 1 (REF) | 0.719 |
| WHO Stage 2 | 0.85 (0.42 – 1.71) |  | 0.74 (0.34 – 1.58) |  |
| WHO Stage 3 | 1.00 (0.49 – 2.10) |  | 0.80 (0.33 – 1.94) |  |
| WHO Stage 4 | 3.25 (1.38 – 7.67) |  | 1.48 (0.34 – 6.51) |  |
|  |  |  |  |  |
| **Provider** |  |  |  |  |
| Provider Type |  |  |  |  |
| Clinical Officer | 1 (REF) | 0.244 | 1 (REF) | 0.598 |
| Nurse | 1.02 (0.57 – 1.83) |  | 0.80 (0.39 – 1.64) |  |
| Medical Officer | 1.53 (0.93 – 2.52) |  | 1.37 (0.68 – 2.73) |  |
|  |  |  |  |  |
| Provider Sex |  |  |  |  |
| Female | 1 (REF) | 0.980 | 1 (REF) | 0.845 |
| Male | 1.01 (0.68 – 1.50) |  | 1.05 (0.61 – 1.49) |  |
|  |  |  |  |  |
| **Interaction** |  |  |  |  |
| Interaction Language |  |  |  |  |
| English | 1 (REF) | 0.197 | 1 (REF) | 0.268 |
| Bemba | 1.07 (0.63 – 1.83) |  | 0.99 (0.50 – 1.96) |  |
| Nyanja | 0.71 (0.46 – 1.12) |  | 0.67 (0.38 – 1.17) |  |
|  |  |  |  |  |
| Client:Provider Sex Concordance |  |  |  |  |
| Not Concordant | 1 (REF) | 0.374 | 1 (REF) | 0.381 |
| Concordant | 1.20 (0.81-1.78) |  | 1.22 (0.79 – 1.88) |  |
|  |  |  |  |  |
| Time Period |  |  |  |  |
| 01 Aug 2019 – 31 Mar 2020 | 1 (REF) | 0.010 | 1 (REF) | 0.002 |
| 01 Apr 2020 – 30 Sept 2020 | 1.92 (1.02 – 3.63) |  | 2.80 (1.21 – 6.45) |  |
| 01 Oct 2020 – 30 Nov 2021 | 0.89 (0.52 – 1.50) |  | 0.95 (0.51 – 1.75) |  |
|  |  |  |  |  |
| Interaction length, per minute increase | 1.04 (1.01 – 1.08) | 0.015 | 1.05 (1.01 – 1.09) | 0.027 |
|  |  |  |  |  |
| **Facility** |  |  |  |  |
| Facility Type^a^ |  |  |  |  |
| Small Clinic | 1 (REF) | 0.169 | 1 (REF) | 0.011 |
| Medium Clinic | 0.82 (0.50 – 1.36) |  | 0.47 (0.21 – 1.06) |  |
| Large Clinic | 0.75 (0.42 – 1.36) |  | 0.45 (0.20 – 1.01) |  |
| Hospital-Based Clinic | 0.46 (0.23 – 0.92) |  | 0.22 (0.09 – 0.54) |  |
|  |  |  |  |  |
| ART Integration^b^ |  |  |  |  |
| Non-Integrated | 1 (REF) | 0.625 | 1 (REF) | 0.302 |
| Integrated | 0.90 (0.58-1.38) |  | 0.75 (0.43 – 1.30) |  |
|  |  |  |  |  |
| Client:Provider Ratio^c^, per 50 client increase | 1.0 (0.96-1.04 | 1.0 | 1.05 (1.0 – 1.11) | 0.051 |

^a^Small Clinic – 0-2,500 clients; Medium Clinic – 2,500-10,000 clients; Large Clinic - >10,000 clients; Hospital-based clinic – Outpatient clinic based at facility that also provided inpatient hospital services

^b^ Non-integrated - ART services provided during standalone clinic session; Integrated – ART services integrated with other primary care services

^c^Number of clients in a facility clinic population over number of providers offering ART services at the at facility, averaged quarterly

**REFERENCES**

1. Zou GY, Donner A. Extension of the modified Poisson regression model to prospective studies with correlated binary data. Stat Methods Med Res. 2013;22(6):661-70.
2. Yelland LN, Salter AB, Ryan P. Performance of the modified Poisson regression approach for estimating relative risks from clustered prospective data. Am J Epidemiol. 2011;174(8):984-92.
3. Zou G. A modified poisson regression approach to prospective studies with binary data. Am J Epidemiol. 2004;159(7):702-6.
4. Vittinghoff E, Glidden DV, Shiboski SC, McCulloch CE. Regression Methods in Biostatistics: Linear, Logistic, Survival, and Repeated Measures Models. New York, NY: Springer-Verlag; 2012.
